# Surface Modification and Enhancement of Ferromagnetism in BiFeO_3_ Nanofilms Deposited on HOPG

**DOI:** 10.3390/nano10101990

**Published:** 2020-10-09

**Authors:** Shikhgasan Ramazanov, Dinara Sobola, Farid Orudzhev, Alexandr Knápek, Josef Polčák, Michal Potoček, Pavel Kaspar, Rashid Dallaev

**Affiliations:** 1 Faculty of Physics, Dagestan State University, Makhachkala, St. M. Gadjieva 43-a, 367015 Makhachkala, Russia; ramazanv@mail.ru; 2 Department of Physics, Faculty of Electrical Engineering and Communication, Brno University of Technology, Technicka 2848/8, 61600 Brno, Czech Republic; sobola@vutbr.cz (D.S.); xdalla03@stud.feec.vutbr.cz (R.D.); 3 Central European Institute of Technology, Brno University of Technology, Purkyňova 123, 612 00 Brno, Czech Republic; josef.polcak@ceitec.vutbr.cz (J.P.); michal.potocek@ceitec.vutbr.cz (M.P.); 4 Department of Inorganic Chemistry and Chemical Ecology, Dagestan State University, Makhachkala, St. M. Gadjieva 43-a, 367015 Makhachkala, Russia; farid-stkha@mail.ru; 5 Institute of the Scientific Instruments of the Czech Academy of Sciences v.v.i., Královopolská 147, 61264 Brno, Czech Republic; knapek@isibrno.cz; 6 Institute of Physical Engineering, Faculty of Mechanical Engineering, Brno University of Technology, Technicka 2896/2, 61669 Brno, Czech Republic

**Keywords:** BiFeO_3_, atomic layer deposition, perovskite structure, graphite surface, ferromagnetic properties

## Abstract

BiFeO_3_ (BFO) films on highly oriented pyrolytic graphite (HOPG) substrate were obtained by the atomic layer deposition (ALD) method. The oxidation of HOPG leads to the formation of bubble regions creating defective regions with active centers. Chemisorption occurs at these active sites in ALD. Additionally, carbon interacts with ozone and releases carbon oxides (CO, CO_2_). Further annealing during the in situ XPS process up to a temperature of 923 K showed a redox reaction and the formation of oxygen vacancies (V_o_) in the BFO crystal lattice. Bubble delamination creates flakes of BiFeO_3-x_/rGO heterostructures. Magnetic measurements (M–H) showed ferromagnetism (FM) at room temperature M_s_ ~ 120 emu/cm^3^. The contribution to magnetization is influenced by the factor of charge redistribution on V_o_ causing the distortion of the lattice as well as by the superstructure formed at the boundary of two phases, which causes strong hybridization due to the superexchange interaction of the BFO film with the FM sublattice of the interface region. The development of a method for obtaining multiferroic structures with high FM values (at room temperature) is promising for magnetically controlled applications.

## 1. Introduction

Recently, the demand for the use of multiferroics in various fields and products of nanoelectronics has been growing steadily. One of the most interesting compounds in the form of a thin film is bismuth ferrite (BFO) because of its electric [1], magnetic [2], piezo [3,4], ferroelectric [5], dielectric [6], memristive [7,8] and optical [9,10,11,12] properties. In addition, BiFeO_3_/reduced graphene oxide composites have excellent photocatalytic characteristics due to improved light absorption, an increase in the number of reactive centers and a low rate of recombination of electron–hole pairs [13]. In particular, bismuth ferrite films are of great interest as a material capable of large energy density accumulation (roughly ~ 70 J·cm^−3^) [14]. This makes BFO/graphene important for application as an electrode material for supercapacitors. Another potential use is proposed by Zhenhua Qiao, et al. [15], where the authors presented a calculation system for graphene adsorbed on the (111) BFO surface. The authors proposed to realize the quantum anomalous Hall effect by the non-contact coupling of graphene with an antiferromagnetic insulator, which provides both the broken symmetry of time reversal and spin-orbit coupling (SOC). In [16], a strong connection between the structure and properties and its reaction to external fields is demonstrated by creating defect centres in graphene-based materials. Some theoretical works [17,18] show that the inclusion of SOC in the calculation induces large orbital magnetic moments and involves switching magnetic anisotropy energy (MAE), apparently not only the orbital moments, but also the spin moments can exhibit significant anisotropy.

Various substrates are used to obtain BFO films, including highly oriented pyrolytic graphite (HOPG). HOPG is a material for graphene manufacture by mechanical peeling. Hyun Wook Shin and Jong Yeog Son [19] presented the Graphene/BFO/HOPG capacitor structure obtained by pulsed laser deposition and showed multiferroic properties, namely, ferroelectricity and ferromagnetism. The substrate temperature in this case was ~ 1073 K, at which it was more likely that additional phases would precipitate alongside the polycrystalline BFO phase [20]. In the work of Jiahua Zhu et al. [21] it was shown that the magnetic field plays a decisive role in limiting the process of interfacial relaxation and thereby enhances the capacitance of the electrode, using an external magnetic field can dramatically increase the capacitance without replacing the material and structural modification. The interest in BFO films is significant in comparison to bulk materials because of its spontaneous mechanisms of switching and energy conservation [22]. There are many techniques for producing BFO films. BFO films are produced by metal –organic chemical vapor deposition (MOCVD) in the temperature range of 500–800 °C [23]. Another method of preparation is pulse-laser deposition (PLD) where the growth temperature is about 650 °C [24]. Another method of magnetron sputtering that enables to obtain BFO films exists, where the growth temperature is about 400 °C [25]. An easier way to obtain high-quality and ultra-thin layers is the atomic layer deposition (ALD) [26,27]. The ALD method is also interesting for its low-temperature (523 K) conformal film growth, where the nanocrystalline BFO layers with ferroelectric polarization could be obtained [28]. It is well known that the nucleation of ALD films of metal oxides on chemically inert sp^2^ carbon surfaces is a very difficult task. It was shown that on untreated sp^2^ carbon surfaces, the nucleation of films can be observed only at lattice defects or the edges of steps [29,30,31,32,33,34]. The heterostructure Bi_25_FeO_40_/rGO was obtained Wang Xingfu et al. [32], where the BFO has phase transitions to the Bi_25_FeO_40_ phase with the predominance of oxygen. Research in this work proposes to carry out annealing in vacuum to achieve the goal of obtaining BFO/graphene. MA Jalil et al. [35], reported a bigger increase in the magnetization of a Bi_25_FeO_40_–rGO nanocomposite obtained by the hydrothermal saturation method than of BiFeO_3_–rGO, the authors attribute this to the suppression of the spin cycloid, a decrease in the size of nanoparticles with the additional release of latent magnetization in the Bi_25_FeO_40_–rGO composite material in comparison with pure BFO, which ultimately increased the Dzyaloshinskii–Moriya interaction. To enhance the magnetic properties, BFO is doped with Ho and Mn atoms, such films were obtained by the traditional sol–gel method, where the maximum magnetization reached values Ms ~ 60 emu/cm^3^ [36]. This enhancement was explained by lattice distortions caused by the difference in ion sizes between the doping agent and the matrix, the emergence of new exchange interactions, and the inhibition of the antiferromagnetic spiral modulated spin structure.

This article presents the first preparation of BFO layers on a HOPG surface with the possibility of its delamination into a graphene sublayer. We found that heat treatment in high vacuum promotes the formation of a BFO/rGO heterostructure and delaminates the near-surface HOPG layers creating local defects with the release of carbon oxides (CO, CO_2_). It is shown that the release of lattices of oxygen creates vacancies that promote the growth of ferromagnetism (FM) in the heterostructure. An additional contribution of the superstructure at the film–substrate interface to the FM of the resulting BFO multiferroic is discussed.

## 2. Experimental Details

A specific type and form of carbon structure is required for use in nanophysics and nanotechnology. In order to obtain a thin, stable and smooth surface of the films, HOPG was used as a substrate. This variant of carbon is produced through the pyrolysis of carbon-containing steam at high temperatures of about 2300 K and subsequent annealing at a temperature of about 3275 K [37]. The resulting material forms an almost pure sp^2^ hybridized hexagonal structure with very strong bonds in the graphene plane and weak bonds between planes, which allows relatively easy separation. In this work, ZYA quality HOPG of 7mm × 7 mm dimensions (purchased from NT-MDT Spectrum Instruments, Moscow, Russia) were used. ZYA brand has a mosaic distribution of 0.8° ± 0.2° and the grain size is up to 10 µm.

**Figure 1:**
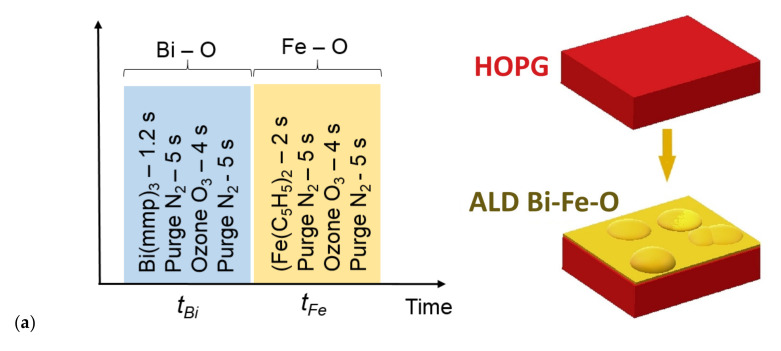

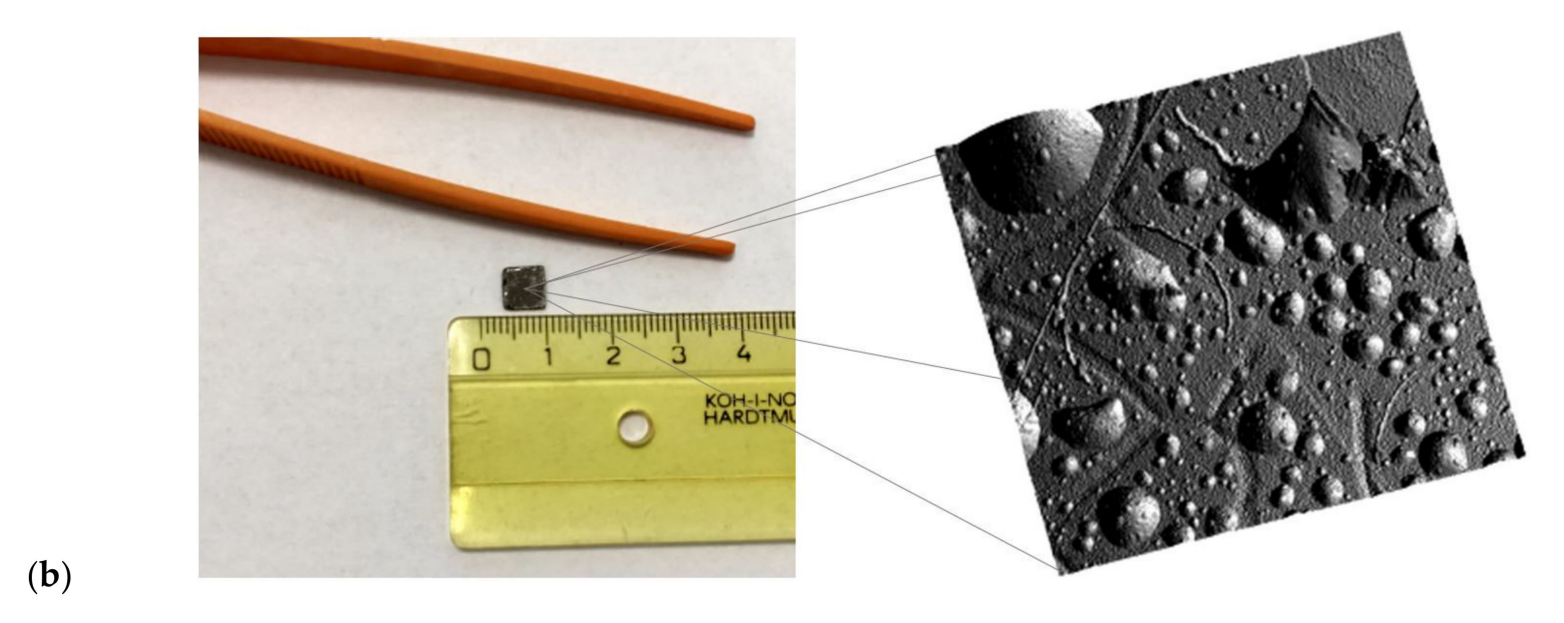
Sequence of atomic layer deposition (ALD) process including sub-cycles for bismuth and iron (**a**), and a sample with dimensions of 7 mm x 7 mm and an SPM-scanned image of a portion of the sample surface (**b**).

To obtain a layer of amorphous bismuth ferrite on the HOPG surface by the ALD method, tris(1-methoxy-2-methyl-2-propoxy) bismuth—C_15_H_33_O_6_Bi or (Bi(mmp)_3_) (Sigma-Aldrich, Schnelldorf, Germany) was used as the Bi-containing organometallic precursor. The evaporation temperature was in the range of 408–418 K. Ferrocene—Fe(C_5_H_5_)_2_ (Sigma-Aldrich, Schnelldorf, Germany) was used as the Fe-containing precursor. The optimal evaporation temperature of ferrocene was 364 K. For oxidation, O_3_ was introduced into the chamber through an ozonizer. A simple method for producing graphene from HOPG by oxidation with ozone and at a low temperature (530 K) was reported by M. J. Webb, et al. [38]. The purge was carried out using carrier gaseous N_2_, with a purity of 99.999%. The substrate was placed at a distance of 4 cm from the inlet. The chamber heated up evenly T = 523 K. The preliminary vacuum in the chamber was 10^−3^ torr. The purge gases at the outlet were held at a constant temperature of 423 K. The process was carried out in 150 sub cycles with each precursor in one technological mode. Figure 1 shows the sequence of the ALD process, where the duration of the *t_Bi_* sub cycle is 15.2 s and the *t_Fe_* sub cycle is 16 s. At the beginning, the BiO_x_ layer and then the FeO_x_ layer were obtained. The thickness of the Bi–Fe–O film was about ~ 40 nm. The result was a modification of the graphite near-surface layer, containing graphene and a small amount of oxygen and hydrogen. The mechanism occurs in our case during the obtaining of a BiO_x_ layer at the initial stage of growth. Figure 1 shows the resulting structure and the sequence of operations for the synthesis of oxides in the BiO_x_–FeO_x_ system on Highly Oriented Pyrolytic Graphite (HOPG).

**Figure 2:**
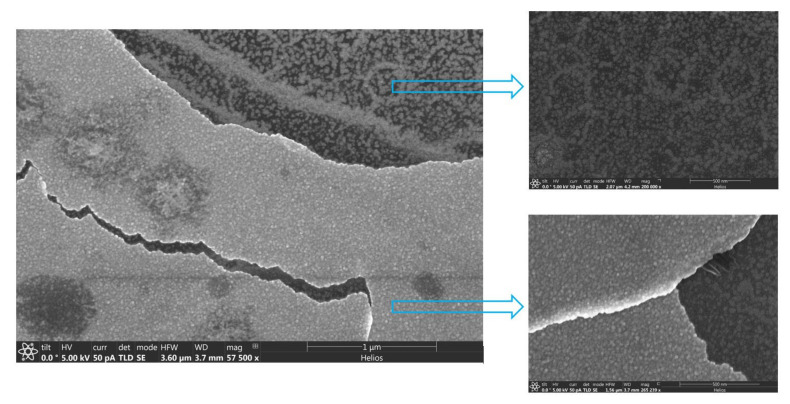
SEM image of the surface area of the bismuth ferrite (BFO)/HOPG structure, the enlarged area on which the delamination of the BFO film from the substrate is visible.

Liu Liwei et al. [39] note that δ-Bi_2_O_3_ can exist at temperatures below 473 K and undergo a sequence of phase transitions δ→β→α with increasing annealing temperature above 473 K. In our case, the bismuth oxide phase is already formed during the growth process, and the iron oxide phase is still in the amorphous state. The occurrence of displacements in the film–substrate interface in the presence of bismuth atoms was observed by Xueyong Pang et al. [40]. Due to the presence of weak Van der Waals bonds between the layers, swelling and delamination (Figure 2) of the surface layers occurs [41,42,43].

The pretreated HOPG substrates and the resulting BFO/HOPG structure were studied using scanning electron microscopy (SEM) (Helios, FEI, Brno, Czech Republic). The surface of the samples was studied by Scanning Probe Microscopy (SPM). Using the time-of-flight secondary ion mass spectrometry on TOF-SIMS^5^ set-up (IONTOF, Muenster, Germany), a qualitative analysis of the sections of the samples in depth was carried out, and a distribution of Bi, Fe, C elements on the surface was performed. Then, the X-ray photoelectron spectroscopy (XPS) (AXIS SupraTM, Kratos Analytical Ltd, Manchester, UK) method was used to study the binding energy of the constituent components with the possibility of in situ heating to a temperature of 923 K.

## 3. Results and Discussion

### 3.1. Secondary-Ion Mass Spectrometry (SIMS) analysis

**Figure 3:**
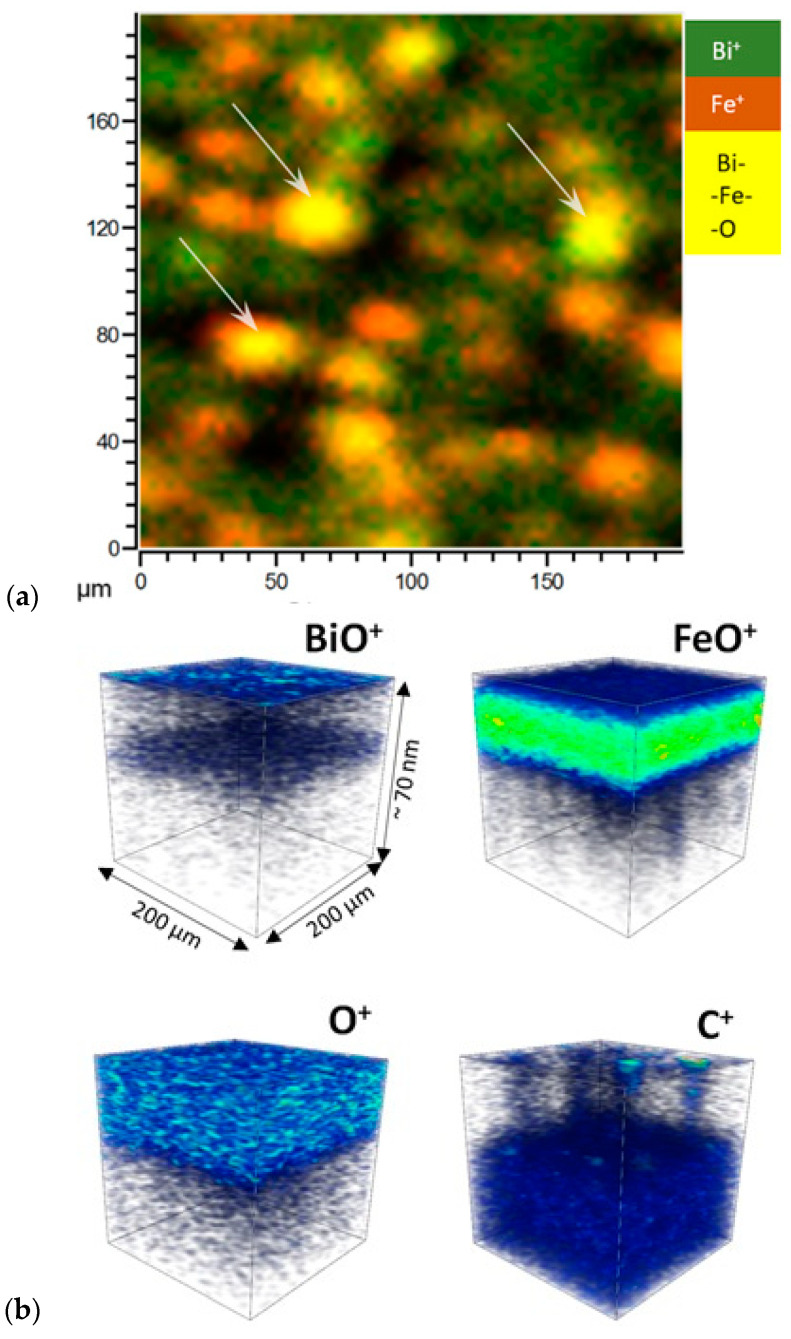
SIMS of the sample surface: composition of the components Fe, Bi, C and their combined image (**a**), an the combined image demonstrates the presence of BFO phase obtained in the process of the film deposition (**b**).

Depth profiling was performed by secondary-ion mass spectrometry (SIMS) and using a Bi^+^ 30 kV analysis beam of 200 μm^2^ area used in imaging mode, and O_2_^+^ 500 V for sputtering. The O_2_^+^ beam also have a signals intensity enhancing effect. The analyses were carried out at TOF-SIMS^5^ set up (IONTOF). The resulting relative concentrations of Bi, Fe are presented in the figure. Yellow sections in the picture with all the compounds of Figure 3 indicate the partially formed bismuth ferrite at 523 K. Green sections indicate the amount of bismuth in the near-surface region. Figure 3 shows SIMS images of the components Fe^+^, Bi^+^, C^+^ and their RGB composite image of a thermally untreated sample.

Figure 3a shows the image of the etched area by detecting Bi^+^ and Fe^+^ ions, the arrows show the places where these elements were mixed prior to the BFO phase: it is interesting that this phase was mainly formed on the surface of more exfoliated film sections, probably associated with oxidative reactions with the partial emission of carbon oxides. In the near-surface region, where the amount of Bi^-^ prevails (green areas of Figure 3), the structure of Bi_1+x_FeO_3_ (x > 1) is formed [44]. There is a number of formal oxidation states displayed by bismuth in its oxides, in particular 3^+^ (Bi_2_O_3_) and 5^+^ (Bi_2_O_5_) (and mixed valence states 3/5^+^ (Bi_2_O_4_, Bi_4_O_7_)). In addition to these phases, the substoichiometric phases BiO, Bi_2_O_2.33_ and Bi_2_O_2.75_ are also possible, but usually as impurity phases in Bi_2_O_3_. In addition, bismuth tends to be reduced to a metallic state when heated or exposed to radiation [45]. Thus, it is expedient to form a first layer with ALD BiO_x_ for homogeneous mixing with the FeO_x_ phase. As can be seen in Figure 3b, part of the BiO_x_ is located in the near-surface region, which is probably due to the activity of bismuth to diffuse to the surface [46].

Delamination also confirms the fact that the observed spherical elements of the surface topography are hollow and do not represent compounds of a different composition, except for the possibility of forming a gas phase from adjacent compounds (CO, CO_2_, etc.). During the film deposition, ozone is introduced into the chamber; it oxidizes the surface of HOPG and forms nanobubbles. It was also noted by Sobola et al. that the oxidation of HOPG with HNO_3_ clearly forms bubbles over the entire surface [47]. Thus, it can be noted that surface swelling is due to the oxidation process. Oxygen in the structure is unevenly distributed, and it can be seen from Figure 3b O^+^ that there are areas with its deficiency. Figure 3b FeO^+^ shows that iron oxide is also partially formed at the synthesis stage.

### 3.2. X-ray Photoelectron Spectroscopy (XPS) Analysis

**Figure 4:**
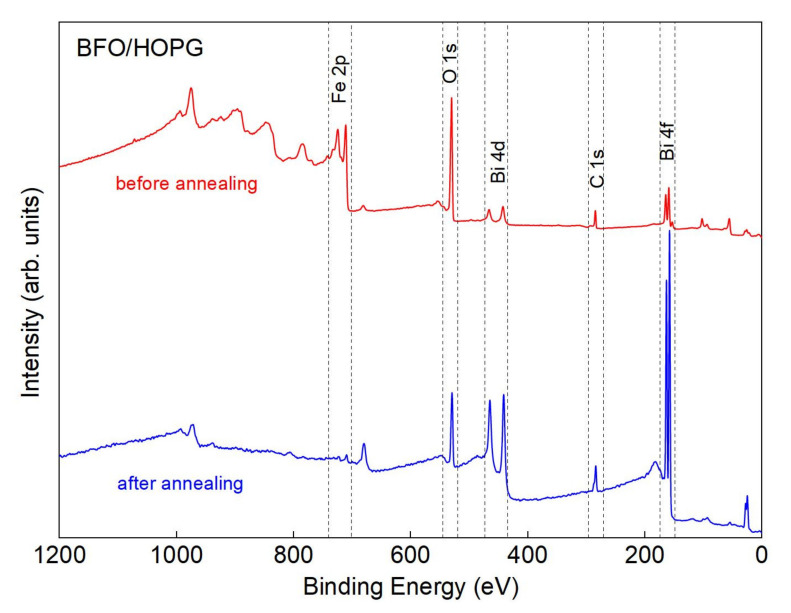
XPS of the BFO/HOPG sample, below—before annealing, above—after annealing at a temperature of 923 K. The main peaks characterizing the compounds formed during the synthesis are noted.

To understand the reactions occurring between the components of the film and the substrate, the samples were studied by XPS with the possibility of in situ heating. Annealing lasted for 5 min for every used temperature. This study was conducted using the tool AXIS SupraTM (Kratos Analytical Ltd, Manchester, UK), vacuum, which during the measurement was about 2 × 10^−8^ torr. The sensitivity of this method is up to 5 nm. XPS spectra were taken several times from both processed and unprocessed areas of the samples. We observed only a slight difference in the components proportion, which can be caused by a non-homogenous distribution of bulking and delaminated areas. The type of chemical bonding according to peak shapes is the same over the whole surface. The size of the investigated region was 300 × 700 μm. Annealing was carried out for approximately one minute. Detailed information about peaks was obtained by the subtraction of Shirley background using CasaXPS software. Figure 4 shows the XPS spectra of the sample before and after annealing.

Figure 4 shows that, prior to annealing, BiO_x_ is formed in the ALD process (523 K), in addition, the bismuth precursor C_15_H_33_O_6_Bi consists of hydrocarbon ligands. At the initial stage, matrix carbon, as a result of interaction with oxygen and BiO_x_, partially forms a bismuth oxycarbonate compound (BiO)_2_CO_3_ in the film. Considering the SIMS results and the fact that bubbles [48] begin to form on the film surface, the decomposition of bismuth oxycarbonate is probably the next step:

(BiO)_2_CO_3_ → Bi_2_O_3_ + CO_2_ (1)

In addition, excess bismuth in the film creates the Bi_1+x_FeO_3_ structure (at x > 1) (green spaces in Figure 3). Then, during annealing, point defects in the form of oxygen vacancies V_o_ and bismuth V_Bi_′′′ are formed in the resulting structure, due to the volatilization of bismuth, which leads to a change in the valence of iron. This can be written according to the notation Kröger–Vink [49]:

2Bi_Bi_ + 3O_O_ → 2V_Bi_′′′ + 3V_Ö_ + Bi_2_O_3_ (2)

(3) $$2{\mathrm{Fe}}_{\mathrm{Fe}}+O_{O}\to 2{\left({\mathrm{Fe}}_{{}_{{\mathrm{Fe}}^{3+}}}^{2+}\right)}^{\prime }+V_{\ddot{O}}\;+\;1/2O_{2}$$

**Figure 5:**
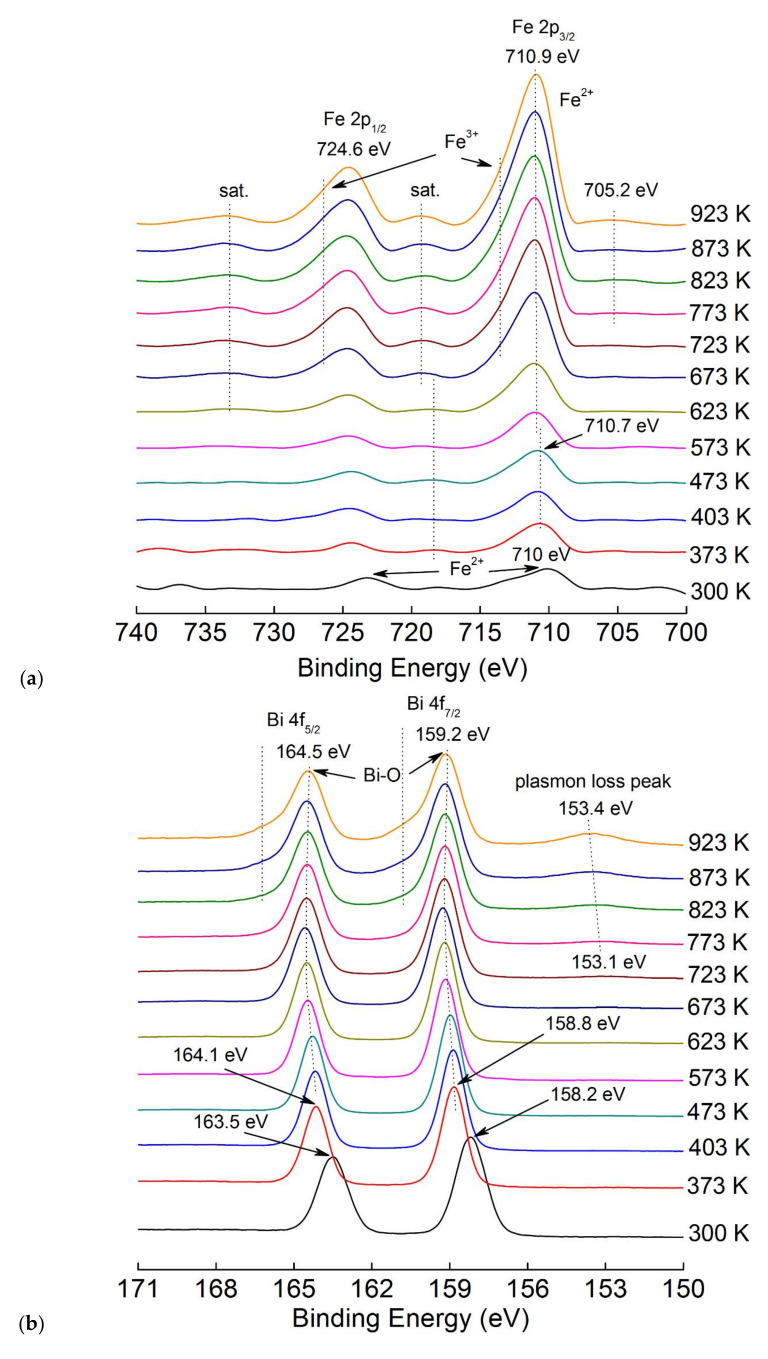

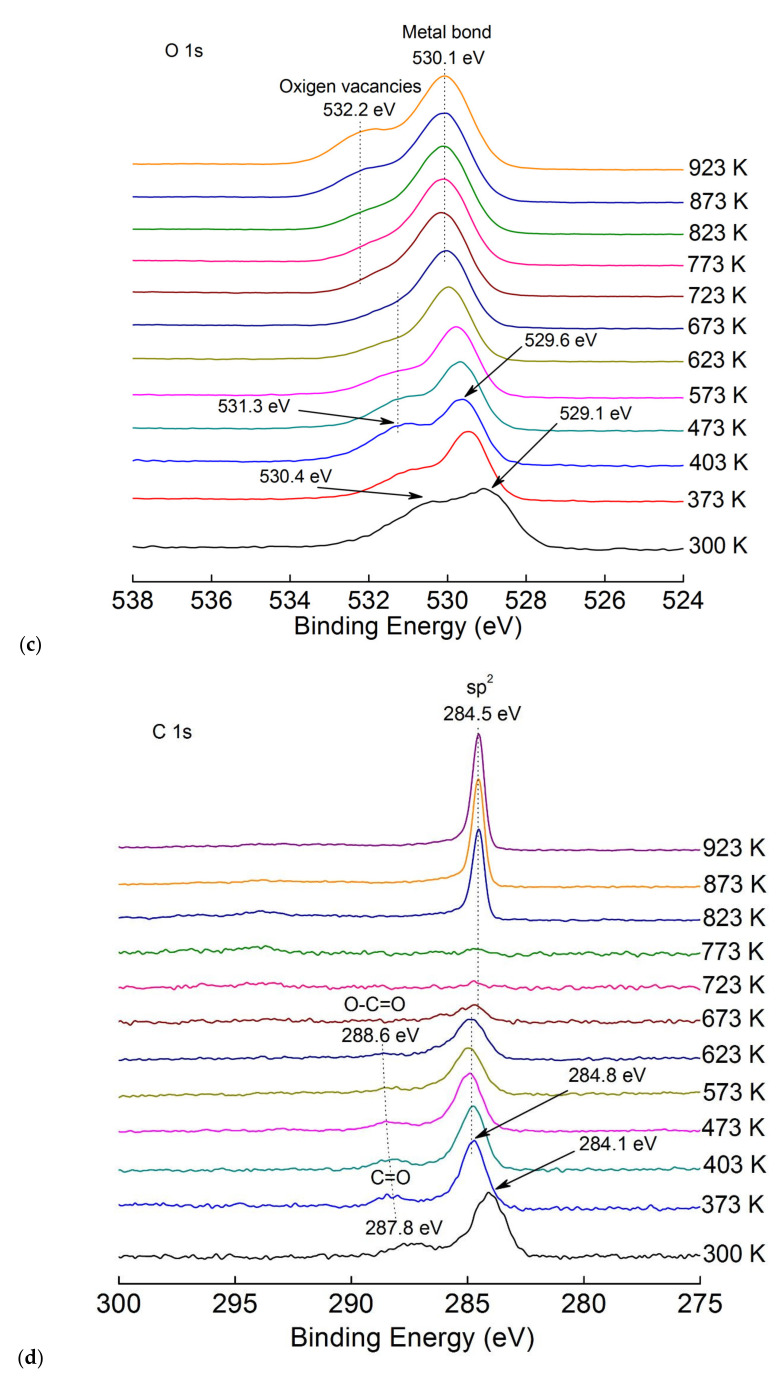
XPS BFO/HOPG structure: (**a**) high-resolution Fe 2p spectra; (**b**) high-resolution Bi 4f spectra; (**c**) high-resolution O 1s spectra; and (**d**) high-resolution C 1s spectra.

For a detailed understanding of the reactions that occur during heat treatment in high vacuum between the components of the film and the substrate, XPS studies of the sample were carried out with the possibility of in situ annealing from 300 to 923 K (Figure 5).

After calibrating the spectra at the C1s position corresponding to the C–C bond (284.8 eV), the positions of the high-resolution Fe, Bi, and O peaks were evaluated. Detailed information about peaks was obtained by the subtraction of the Shirley background using CasaXPS software. Normalized spectra are given in Figure 5a–d for a comparative analysis at different annealing temperatures. The XPS spectra of Fe has 2p_3/2_ and 2p_1/2_ peaks, which can be fitted as two peaks for Fe^2+^ and Fe^3+^ (Figure 5a). As can be seen from Figure 5a, before annealing (300 K), the spin–orbit splitting already occurs on the XPS Fe2p spectrum, which indicates the initial stage of the formation Fe–O crystallization. Bi^3+^ oxidation state is observed at peaks 4f_7/2_ and 4f_5/2_ located at 158.2 eV and 163.5 eV (Figure 5b). According to the literature [50,51], the largest peak of oxygen near 529.1 eV could be assigned to oxygen in the BFO structure and the oxygen peak at 530.4 eV corresponds to dangling bonds of oxygen (Figure 5c). Peaks at the binding energies of 710 and 724 eV represent a doublet of Fe 2p_3/2_ and Fe 2p_1/2_, which becomes more noticeable as a result of the gain of the spin–orbit interaction. In this case, the energy of the spin–orbit splitting of the doublet is ~ 13.7 eV, in contrast with the theoretical value (ΔFe2p) of 13.6 eV for Fe_2_O_3_ [51,52]. In addition, the peaks of Fe 2p_1/2_ and Fe 2p_3/2_ at high temperatures are shifted by 0.9 eV and 0.5 eV, respectively. The redshift suggests a certain electronic interaction between the BFO and rGO sheet [53] due to difference in the electronic configuration of Fe^2+^ (d_6_) and Fe^3+^ (d_5_) ions. Satellite peaks are very often used to determine the chemical state of iron. As can be seen from Figure 5a, all satellite peaks appear at 8–8.5 eV higher binding energy than the corresponding main peaks in the experimental spectra of Fe2p. This result convincingly indicates the existence of only the Fe^3+^ state for all samples, since the satellite peaks arise at a binding energy of 6 eV higher than the corresponding main peak for the Fe^2+^ state [54].

The narrow-band spectra of Bi4f level, characterized by the presence of a doublet peak consisting of Bi4f_7/2_ (158.2 eV) and Bi4f_5/2_ (163.5 eV) are presented in Figure 5b. More detailed information on the variation of Bi 4f peaks depending on the annealing temperature is given in Table S1. Although a shift in the position of the peaks towards higher binding energies (159.2 and 164.5 eV, respectively) is observed with increasing temperature, the energy of the spin–orbit splitting of the Bi4f doublet remains unchanged (5.3 eV), which is in good agreement with theoretically calculated values (5.31 eV) [51]. The positions of the Bi4f peaks confirms the presence of the Bi^3+^ state in the sample and are identified as a signal from Bi–O bonds [53,55], which is manifested on XPS at an annealing temperature of 773 K. In this regard, the wide peak in the region of 532.2 eV in the O1s spectrum (Figure 5c), which is detected at the annealing temperature of 673 K, can be attributed to superoxide bonds in the BFO film. On Bi4f XPS, a gradual shift of Bi4f peaks is observed as the annealing temperature increases from 373 K to the high-energy region (starting from 164.1 eV for 4f_5/2_ and 158.8 eV for 4f_7/2_), further from the annealing temperature of 723 K to 923 K, the spectra show a small shift into the region of lower binding energies. This effect can be associated with the sequence of reactions 2,3. In the region of 153.1–153.4 eV, a plasmon peak appears, probably associated with charging effects [56] and the formation of the BFO phase with peaks in the region of 4f_5/2_ = 164.5 eV and 4f_7/2_ = 159.2 eV. With an increase in the heat treatment temperature to 623–673 K, the energy of spin–orbit splitting of f Bi electron gradually shifts to the region of high energies. This could be due to a change in the valence state of Bi^3+^→Bi^5+^, and with a further increase from 673 K the reverse shifts to Bi^3+^ occurs [57]. Additional oxygen ions are released by increasing the ratio Fe^3+^/Fe^2+^ at an annealing temperature ≥ 623 K (Figure 5a). More detailed information on the change in the quantitative ration of Fe^3+^/Fe^2+^ depending on the annealing temperature is given in Table S1.

Two regions are clearly observed in the narrow-band spectrum of C1s. They are characterized by a small chemical shift depending on the temperature at binding energies of about 284 eV and 288 eV, respectively [58]. The broad peak with a binding energy of about 284.5 eV can be attributed to the non-oxygen sp^2^ carbon C=C, which represents the structure of HOPG graphene layers [59]. The presence of a wide shoulder in the region of 285 eV indicates the bonding of carbon atoms with oxygen in hydroxyl (C–OH) or epoxy (C–O) functional groups located in the basal plane of graphene sheets, responsible for the presence of defects in the plane [60]. A peak in the region of about 287.8 eV characterizes the bonds of carbon atoms in carbonyl groups (>C=O) [61], corresponding with COOH functional groups, usually located at the edges of graphene sheets [62]. After 823 K, only one region remains in the spectrum characterizing the non-oxygen sp^2^ carbon bond C=C, which indicates the deoxygenation of the delaminated surface of graphene-like HOPG layers [63].

Figure 5d shows the peak associated with the formation of C=O (at annealing temperatures up to 623 K), as well as its transitions to O–C=O in the region of 288.8–288.6 eV with increasing annealing temperature. It was also observed during the formation of a thin layer of iron with the self-organization of the Fe_2_O_3_ phase on the HOPG surface [64]. This is likely due to the catalytic properties of the iron oxide itself [65], The sp^2^ intensity becomes almost invisible in the spectrum during annealing at 673–773 K. This temperature range is also associated with the formation of the iron carbonate phase FeCO_3_ [66]. This is due to the formation of a BFO film in the crystalline phase and the initial stage of carbon reduction in the near-surface region. At an annealing temperature greater than 823 K, a graphene layer with a cohesion energy of 284.5 eV is formed on the lower surface of the already formed BFO film (Figure 5b). More detailed information on the change in the C1 peaks depending on the annealing temperature is given in Table S1.

Peaks for the Fe–O and Bi–O bonds are around 530.1 eV and 531.8 eV, respectively, for all temperatures [67,68]. Bi_2_O_3_ exists in several polymorphic modifications, and also as a material with oxygen vacancies, such as Bi_2_O_2.33_ [69]. The peak near 531.3 eV grows up to 403 K, then decreases and disappears, which indicates the formation of oxygen vacancies [70] and their growth with the release of CO_2_ [71]. At the beginning, the FeO_x_ amorphous phase turns into FeCO_3_ with increasing annealing temperature, and then it partially converts into the Fe_3_O_4_ and into the BFO phases (Equation (4)):

3FeCO_3_ → Fe_3_O_4_ + CO + 2CO_2_ (4)

The final reaction in the solid-state process with the formation of BFO and carbon could be continued as in Equation (5):

3Bi_2_O_3_ + 2Fe_3_O_4_ + CO → 6BiFeO_3_ + C (5)

In the O1s spectrum in the region of ~ 532.2 eV, there is a wide peak characterizing the surface defects in the BFO phase corresponding to V_o_ [72]. From the XPS analysis, it can be seen that when annealed in vacuum, bismuth corbanate or bismuth oxide is not reduced to metallic, but forms a triple compound with iron and oxygen, possibly due to the formation of oxygen and bismuth vacancies [73]. More detailed information on the change in O1 peaks depending on the annealing temperature is given in Table S1. It can be assumed that obtaining Bi–O as the first layer and then Fe–O as the retaining layer with further annealing in high vacuum forms a three-component structure BiFeO_3_.

### 3.3. Vibrating Sample Measurement (VSM) Analysis

Magnetic measurements were carried out using a vibrating magnetometer (Cryogen-Free High Field Measurement System from Cryogenic Limited, London, United Kingdom) in which a linear magnetometer vibrates a sample mounted on a rod. A magnetic field (up to 1 Tesla) is created using a superconducting magnet with liquid helium. The sample oscillates with a frequency of 21 Hz near the detector coil, where the induced voltage is amplified and detected, locking time constant at 0.3 s. The measurements were carried out at a low 10 K and a room temperature of 300 K. In order to provide information on the saturation magnetization and coercivity of the samples, hysteresis loops (M–H curves) were constructed. This is done by measuring the magnetization (M) of the sample, depending on the applied magnetic field strength (H) at a fixed temperature. The hysteresis loops are plotted considering the antiferromagnetic (AFM) and weak ferromagnetic (FM) contributions. The M–H magnetic hysteresis loops for the BFO/HOPG samples before and after annealing, measured at 10 K and 300 K, are shown in Figure 5 when the magnetic field is applied in parallel to the sample surface. It is known that BFO exhibits low-temperature ferromagnetic behavior [74], exhibiting hysteresis; one way to verify this observation is to perform a measurement with cooling at low temperatures (10 K). All BFO systems (bulk and thin-film) exhibit weak magnetization due to spin rotation caused by the tilt of the oxygen octahedron, which is characteristic when the temperature drops below Neel.

**Figure 6:**
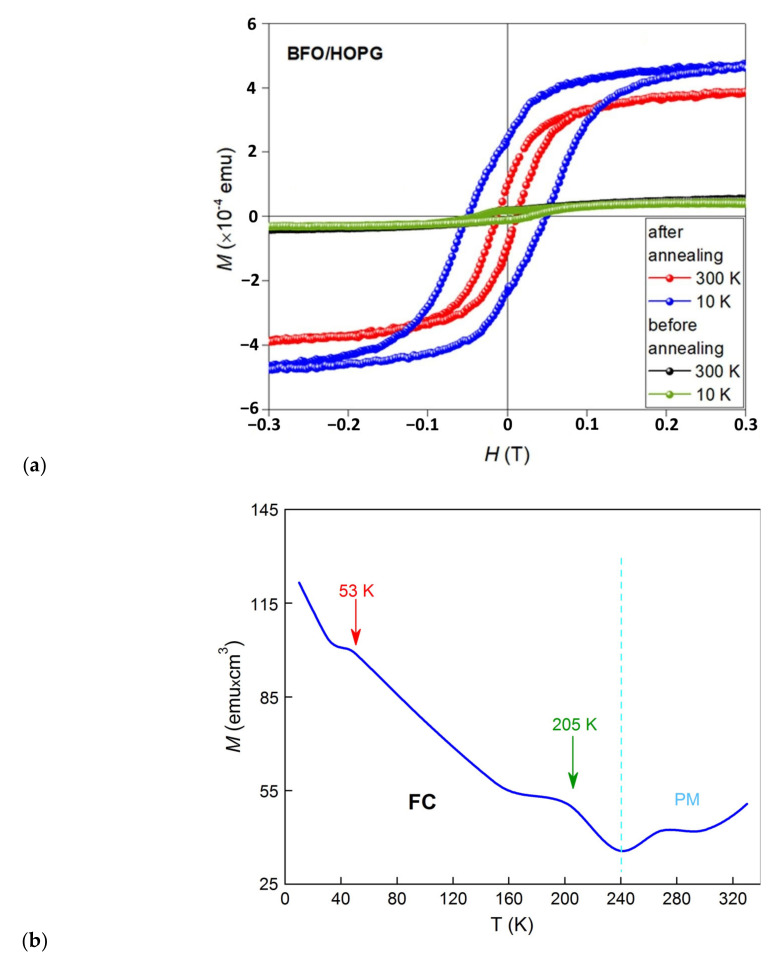
The hysteresis loop of the BFO/HOPG structure of the unannealed and annealed sample at 927 K, M–H dependencies recorded at room (300 K) and at low (10 K) temperatures (**a**); magnetization as a function of temperature measured during cooling in the field 200 Oe (**b**).

As can be seen in Figure 6a, the M–H hysteresis loop of the unannealed BFO/HOPG sample practically does not change at room and low magnetization removal temperatures. A soft ferromagnet-like behavior is observed, probably related to the obtained nanoscale structure, which contributes to the appearance of small spin twisting in the structure of the AFM phase film [75]. It is also partially associated with defective inclusions in HOPG formed at the stage of obtaining the ALD layer (Fe–C bond) [76]. Magnetization as a function of temperature measured upon cooling in a field of 200 Oe (FC) showed several features (Figure 6b): (1) as the temperature is lowered from 300 K to 10 K the magnetization monotonously decreases; a protrusion appears at 53 K characteristic to the spin glass [77]; (2) heterogeneity in the area of 215 K can be attributed to the magnetic blocking mechanism caused by the contention between the thermal energy and the energy of magnetic anisotropy; and (3) an increase in the magnetization in the tail region at 300 K indicates a paramagnetic phase which is characteristic for oxygen vacancies in the BFO film.

In addition, the nature of M–H can be affected by the diamagnetism (DM) of graphene layers in the HOPG, which have different values depending on the direction of the applied external field, where ⊥ ~ 950 (χ × 10^−6^) and ‖ ~ 85 (χ × 10^−6^). At the stage of the ALD process, upon the separation of the surface region and the formation of bubbles, these values are mixed and form an intermediate state, which is also associated with the nanoscale formed crystalline phase of the BFO film. A certain AFM(–FM)/DM heterostructure is formed. It is likely that graphite layers affect the overall magnetization of the system, which prevents the growth of film magnetization at low temperatures (10 K) of the unannealed sample. The electronic subsystem of bonds –C=C, –C=O with the obtained film does not allow the AFM state to twist in the film up to low temperatures (Figure 5). Probably, another factor affecting the overall magnetization of the system is the spin–orbit interaction of the graphene layers on the underside of the exfoliated film, which creates anisotropy by partially “blocking” the crystal field, and suppresses the electron spin moment in the AFM phase of the film. It can be assumed that the magnetic properties of the rGO sublayer compete with the spin magnetic subsystem of the BFO film.

The heat treatment of a BFO/HOPG heterostructure sample at 927 K induces the crystallization of the deposited films of amorphous-like nature. After annealing, the BFO/HOPG structure behaves differently showing a more significant FM character (Figure 6), the magnetization grows, and the coercive force also grows from *H*_c_ = 12 mT to *H*_c_ = 49 mT due to the FM properties of the BFO film (Figure 5). The origin of ferromagnetism in the BFO can be explained by the double exchange interaction. According to the double exchange interaction, the electron transfers from Fe^2+^ to O^2–^ and at the same time, the electrons jump from O^2−^ to Fe^3+^, thereby completing the electron transfer from Fe^2+^ to Fe^3+^. More and more theoretical and experimental works provide evidence that magnetic ordering is closely related to V_o_ [78,79], where V_o_ additionally causes lattice distortion and leads to an increase in FM. The enhancement of the FM state is likely to be a paradox resulting from the competition between the orbital hybridization of Fe_3d_–O_2p_ and the crystalline field of FeO_6_. When the interfacial region is suppressed in one direction at the interface, the lateral MAE increases. In a recently published paper by Pingfan Chen et al. [80], the enhancement of magnetic anisotropy and the violation of orbital symmetry in manganite heterostructures is described at length. Authors Yan-Fei Wu et al. [81] reported the effect of magnetic proximity in the graphene/BFO heterostructure leading to strong Zeeman splitting in graphene with an exchange field of up to hundreds of tesla.

Thus, it can be assumed that the great part of the BFO/HOPG structure magnetization is due to two reasons: the charge redistribution on V_o_ and the contribution of the exchange field of the superlattice at the interface. A new magnetic configuration in the Fe^3+^ –O – Fe^3+^ sublattice in the BFO structure caused by strong hybridization at the film / substrate interface creates a superexchange interaction with the Fe–C superstructure [82]. An additional enhancement of the FM state at the BFO/HOPG interface is likely due to the spin polarization of electrons in the carbon layer, forming a bond between the electrons of the two materials [83]. The calculation of the FM state magnetization from the obtained values: the saturation magnetization at room temperature M_s_ = V/M gives the value of *M*_s_ ~ 120 emu/cm^3^.

## 4. Conclusions

The atomic layer deposition method enables obtaining ultra-thin BFO multiferroic films on the HOPG surface at low temperatures. In the process of synthesis, the HOPG surface is modified and some bubbles and delaminated areas are obtained. According to the TOF-SIMS results, the good miscibility of the BiO_x_ and FeO_x_ components was detected on the HOPG surface. During annealing in vacuum, it was seen that the BFO film self-organizes and carbon is reduced. This two-stage method (preparation and annealing) allows us to design high-quality structures based on multiferroic materials with oxygen vacancies. Magnetic measurements showed a large increase in the magnetization of the sample due to both the oxygen vacancies and superstructure properties formed at the BFO/HOPG interface. At the film–substrate interface, a new magnetic configuration forms in the Fe^3+^–O–Fe^3+^ sublattice of the BFO structure, which causes strong hybridization due to the superexchange interaction with the FM sublattice of the reduced graphene in which iron forms bonds with dangling cores of the –C=C sublayer.

## Supplementary Materials

The following are available online at https://www.mdpi.com/2079-4991/10/10/1990/s1, Table S1: XPS spectra of Fe 2p, Bi 4f, O 1s, C 1s components depending on the annealing temperature.
