# Supplementary material for: Surface Modification and Enhancement of Ferromagnetism in BiFeO3 Nanofilms Deposited on HOPG
Source: Nanomaterials (Basel). 2020 Oct 9;10(10):1990. doi: 10.3390/nano10101990 (PMC7600225; doi:10.3390/nano10101990)
Supplement: Supplementary file 1 [file nanomaterials-10-01990-s001.pdf]

nanomaterials

Supplementary Materials

Surface Modification and Enhancement of Ferromagnetism in $BiFeO_3$ Nanofilms Deposited on HOPG

Shikhgasan Ramazanov [1], Dinara Sobola [2,3,4], Farid Orudzhev [4], Alexandr Knápek [5], Josef Polčák [3,6], Michal Potoček [3,6], Pavel Kaspar [2,*] and Rashid Dallaev [2]

1 Faculty of Physics, Dagestan State University, Makhachkala, St. M. Gadjieva 43-a, 367015 Makhachkala, Russia; ramazanv@mail.ru (S.R.)
2 Department of Physics, Faculty of Electrical Engineering and Communication, Brno University of Technology, Technicka 2848/8, 61600 Brno, Czech Republic; sobola@vutbr.cz (D.S.); xdalla03@stud.feec.vutbr.cz (R.D.)
3 Central European Institute of Technology, Brno University of Technology, Purkyňova 123, 61200 Brno, Czech Republic; josef.polcak@ceitec.vutbr.cz (J.P.); michal.potocek@ceitec.vutbr.cz (M.P.)
4 Department of Inorganic Chemistry and Chemical Ecology, Dagestan State University, Makhachkala, St. M. Gadjieva 43-a, 367015 Makhachkala, Russia; farid-stkha@mail.ru
5 Institute of the Scientific Instruments of the Czech Academy of Sciences v.v.i., Královopolská 147, 61264 Brno, Czech Republic; knapek@isibrno.cz
6 Institute of Physical Engineering, Faculty of Mechanical Engineering, Brno University of Technology, Technicka 2896/2, 61669 Brno, Czech Republic
* Correspondence: kasparp@feec.vutbr.cz; Tel: +420-54114-6009

3.2. X-ray Photoelectron Spectroscopy (XPS) Analysis

Detailed description of XPS spectra of all components depending on the annealing temperature of the obtained structure Fe 2p, Bi 4f, O 1s, C 1s. The deconvolution of the peaks was carried out using Casa XPS software Version 2.3.19PR1.0 (Casa Software Ltd, Wilmslow, UK). The spectra were calibrated by C1s at 284.8 eV and we used Shirley background type and Asymmetric line-shape (LA) of component peaks.

Table S1. XPS spectra of Fe 2p, Bi 4f, O 1s, C 1s components depending on the annealing temperature.

T (K)	Fe 2p

300

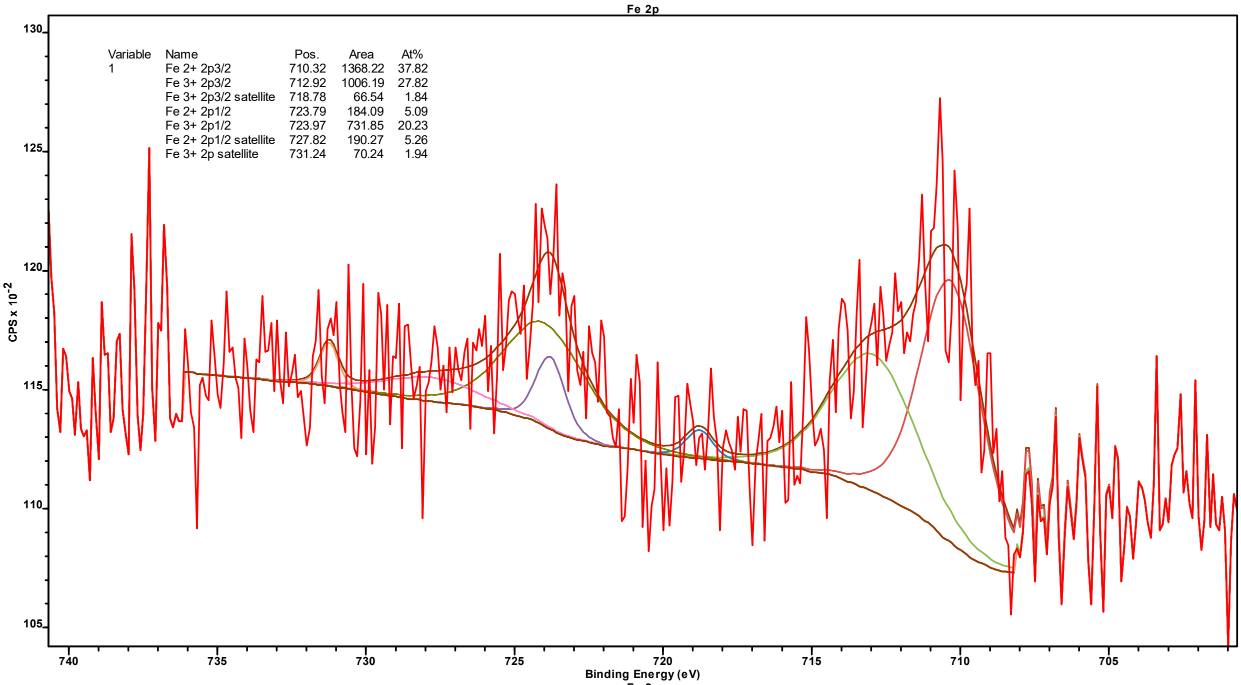
Fe 2p
Variable Name Pos. Area At%
1 Fe 2+ 2p3/2 710.32 1368.22 37.82
Fe 3+ 2p3/2 712.92 1006.19 27.82
Fe 3+ 2p3/2 satellite 718.78 66.54 1.84
Fe 2+ 2p1/2 723.79 184.09 5.09
Fe 3+ 2p1/2 723.97 731.85 20.23
Fe 2+ 2p1/2 satellite 727.82 190.27 5.26
Fe 3+ 2p satellite 731.24 70.24 1.94
CPS x 10-2
Binding Energy (eV)

373

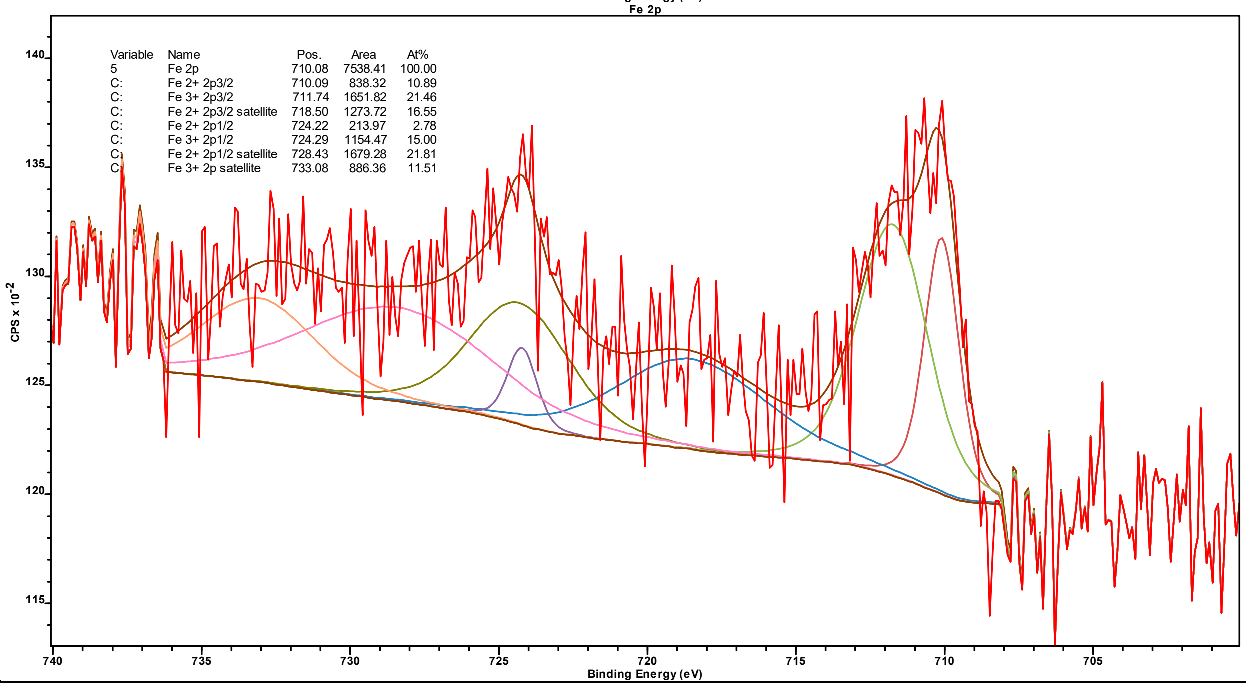
Fe 2p
Variable Name Pos. Area At%
5 Fe 2p 710.08 7538.41 100.00
C: Fe 2+ 2p3/2 710.09 838.32 10.89
C: Fe 3+ 2p3/2 711.74 1651.82 21.46
C: Fe 2+ 2p3/2 satellite 718.50 1273.72 16.55
C: Fe 2+ 2p1/2 724.22 213.97 2.78
C: Fe 3+ 2p1/2 724.29 1154.47 15.00
C: Fe 2+ 2p1/2 satellite 728.43 1679.28 21.81
C Fe 3+ 2p satellite 733.08 886.36 11.51
CPS x 10-2
Binding Energy (eV)

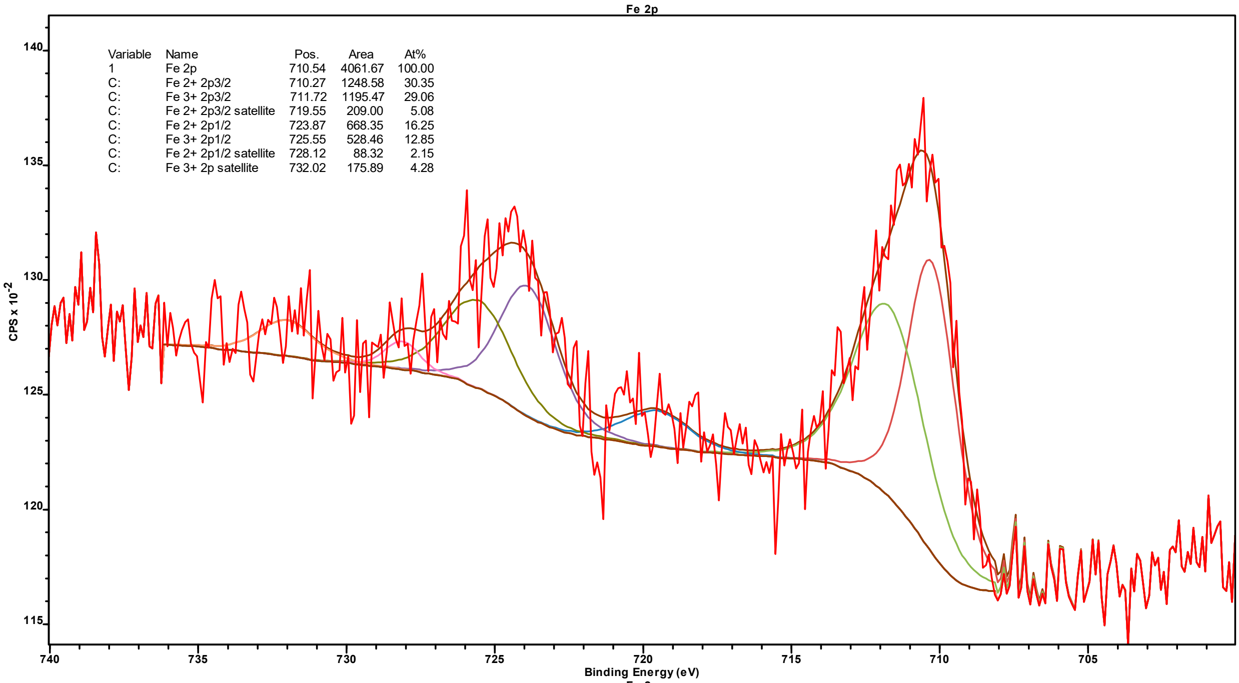
Fe 2p
Variable Name Pos. Area At%
1 Fe 2p 710.54 4061.67 100.00
C: Fe 2+ 2p3/2 710.27 1248.58 30.35
C: Fe 3+ 2p3/2 711.72 1195.47 29.06
C: Fe 2+ 2p3/2 satellite 719.55 209.00 5.08
C: Fe 2+ 2p1/2 723.87 668.35 16.25
C: Fe 3+ 2p1/2 725.55 528.46 12.85
C: Fe 2+ 2p1/2 satellite 728.12 88.32 2.15
C: Fe 3+ 2p satellite 732.02 175.89 4.28
CPS x 10-2
Binding Energy (eV)

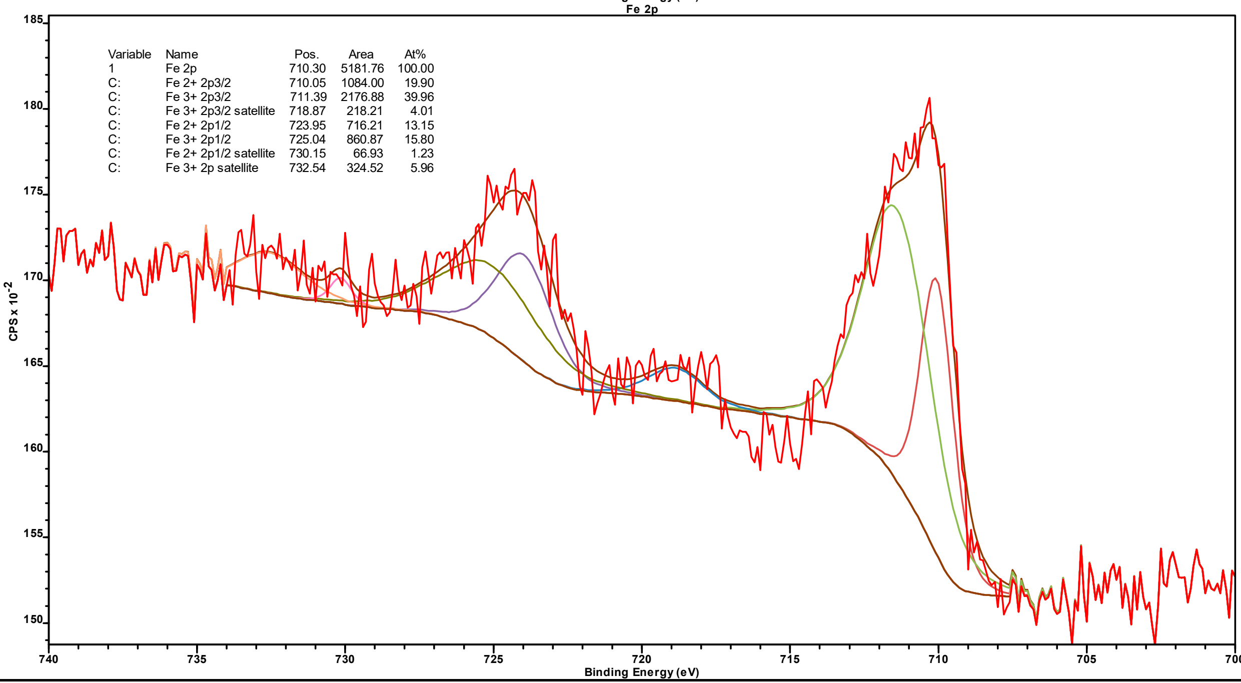
Fe 2p
Variable Name Pos. Area At%
1 Fe 2p 710.30 5181.76 100.00
C: Fe 2+ 2p3/2 710.05 1084.00 19.90
C: Fe 3+ 2p3/2 711.39 2176.88 39.96
C: Fe 3+ 2p3/2 satellite 718.87 218.21 4.01
C: Fe 2+ 2p1/2 723.95 716.21 13.15
C: Fe 3+ 2p1/2 725.04 860.87 15.80
C: Fe 2+ 2p1/2 satellite 730.15 66.93 1.23
C: Fe 3+ 2p satellite 732.54 324.52 5.96
CPS x 10-2
Binding Energy (eV)

573

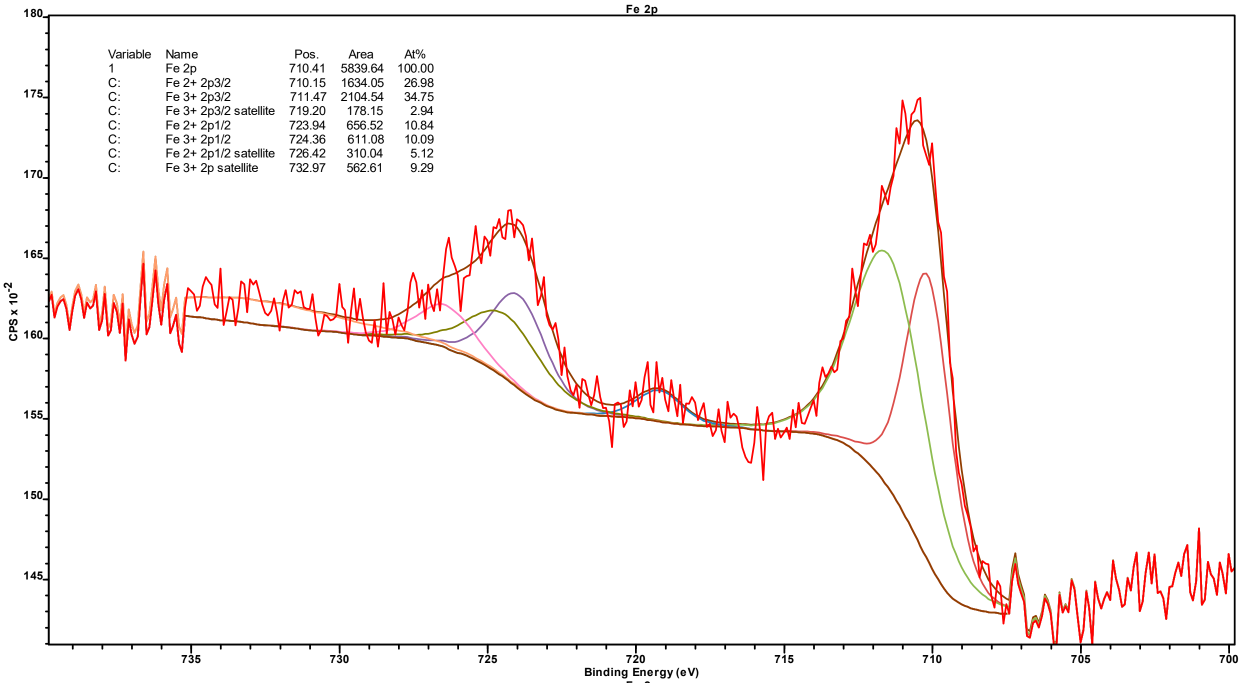
Fe 2p
Variable Name Pos. Area At%
1 Fe 2p 710.41 5839.64 100.00
C: Fe 2+ 2p3/2 710.15 1634.05 26.98
C: Fe 3+ 2p3/2 711.47 2104.54 34.75
C: Fe 3+ 2p3/2 satellite 719.20 178.15 2.94
C: Fe 2+ 2p1/2 723.94 656.52 10.84
C: Fe 3+ 2p1/2 724.36 611.08 10.09
C: Fe 2+ 2p1/2 satellite 726.42 310.04 5.12
C: Fe 3+ 2p satellite 732.97 562.61 9.29
CPS x 10^-2
Binding Energy (eV)

623

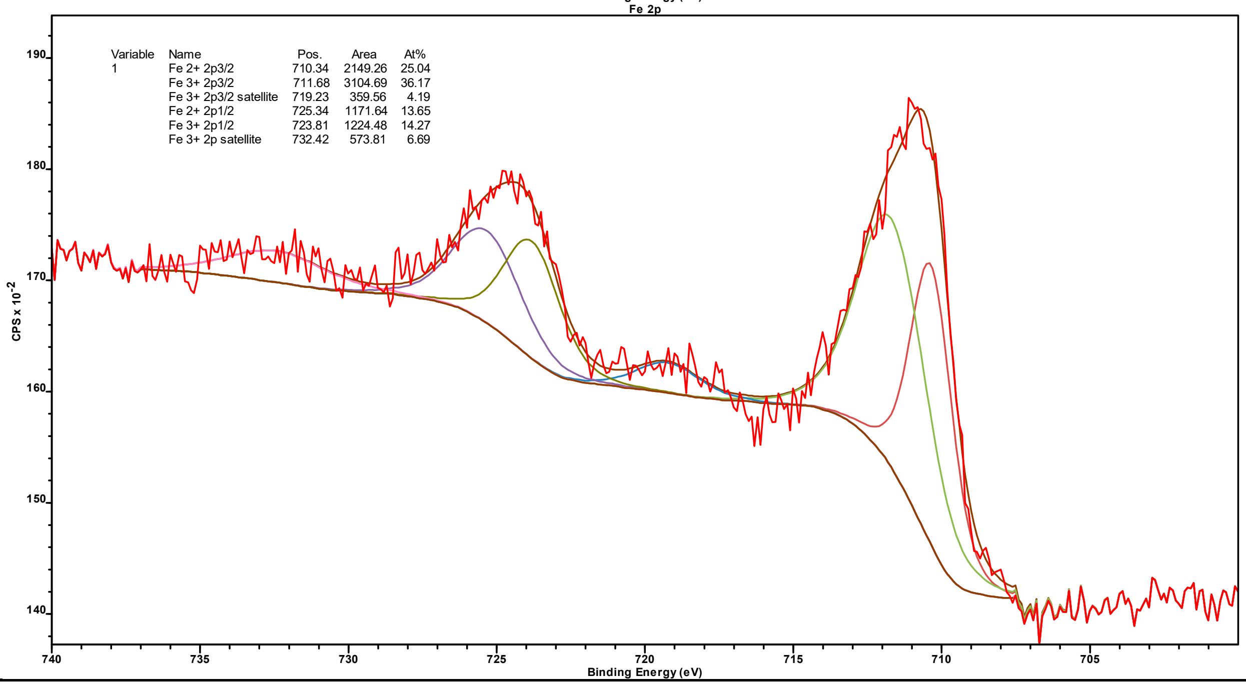
Fe 2p
Variable Name Pos. Area At%
1 Fe 2+ 2p3/2 710.34 2149.26 25.04
Fe 3+ 2p3/2 711.68 3104.69 36.17
Fe 3+ 2p3/2 satellite 719.23 359.56 4.19
Fe 2+ 2p1/2 725.34 1171.64 13.65
Fe 3+ 2p1/2 723.81 1224.48 14.27
Fe 3+ 2p satellite 732.42 573.81 6.69
CPS x 10^-2
Binding Energy (eV)

673

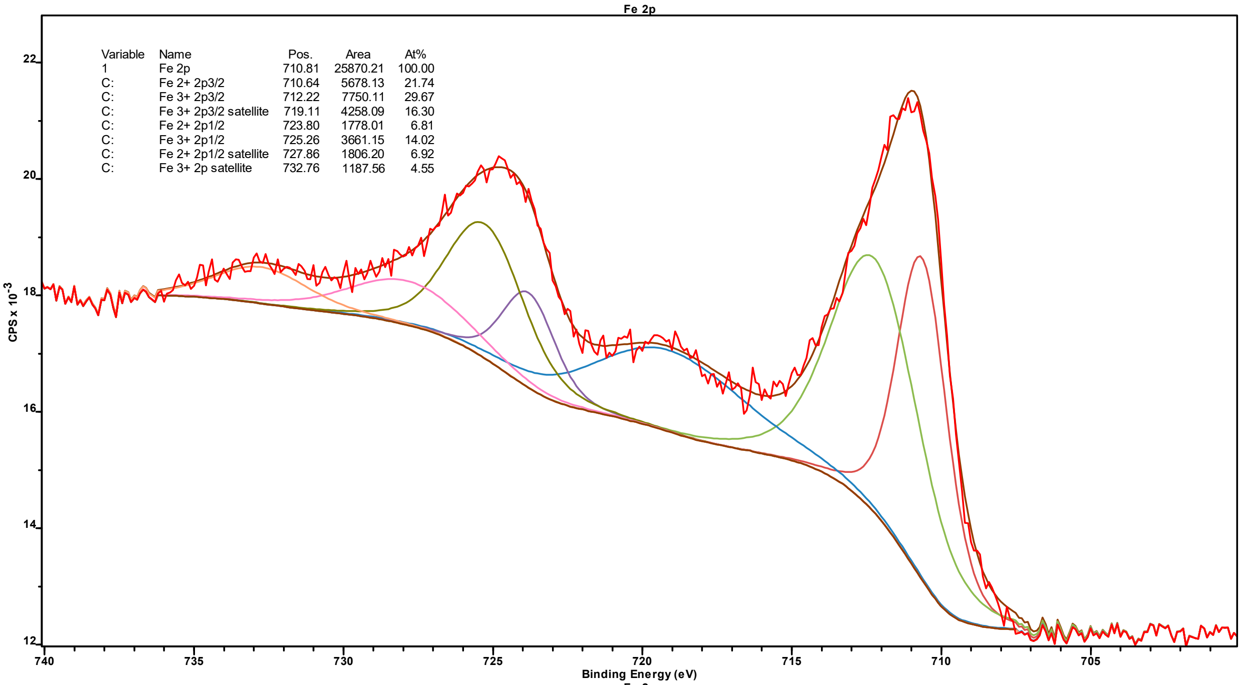
Fe 2p
Variable Name Pos. Area At%
1 Fe 2p 710.81 25870.21 100.00
C: Fe 2+ 2p3/2 710.64 5678.13 21.74
C: Fe 3+ 2p3/2 712.22 7750.11 29.67
C: Fe 3+ 2p3/2 satellite 719.11 4258.09 16.30
C: Fe 2+ 2p1/2 723.80 1778.01 6.81
C: Fe 3+ 2p1/2 725.26 3661.15 14.02
C: Fe 2+ 2p1/2 satellite 727.86 1806.20 6.92
C: Fe 3+ 2p satellite 732.76 1187.56 4.55
CPS x 10-3
Binding Energy (eV)

723

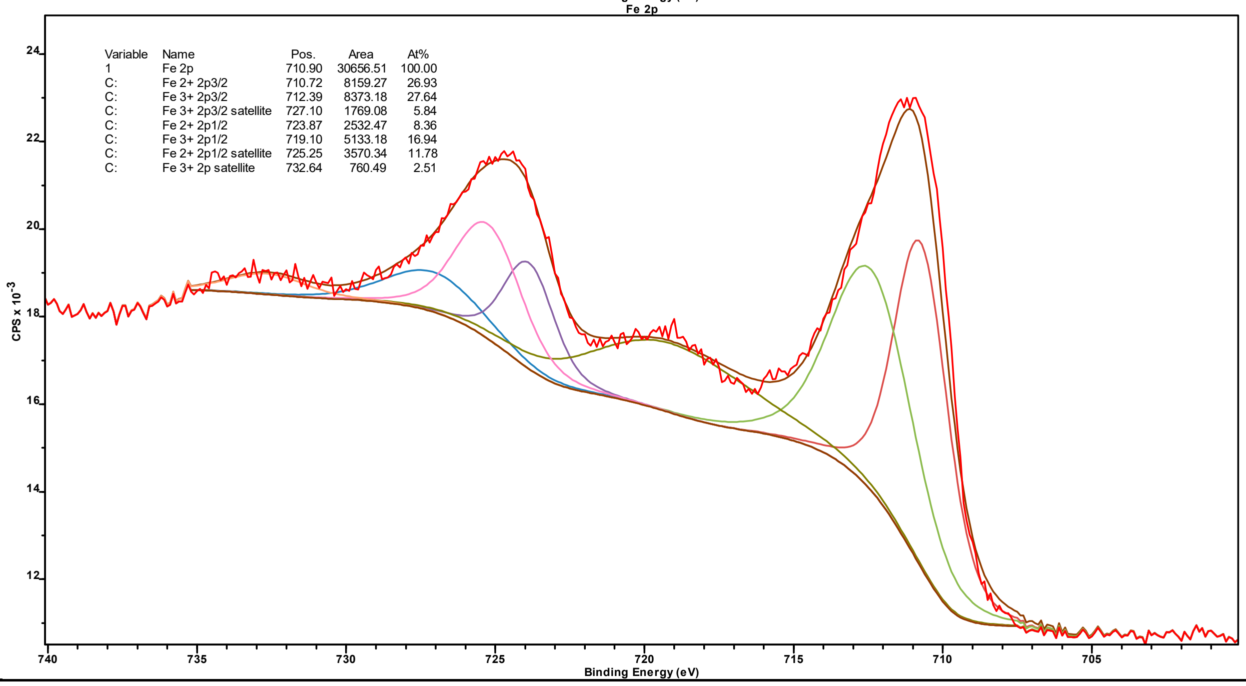
Fe 2p
Variable Name Pos. Area At%
1 Fe 2p 710.90 30656.51 100.00
C: Fe 2+ 2p3/2 710.72 8159.27 26.93
C: Fe 3+ 2p3/2 712.39 8373.18 27.64
C: Fe 3+ 2p3/2 satellite 727.10 1769.08 5.84
C: Fe 2+ 2p1/2 723.87 2532.47 8.36
C: Fe 3+ 2p1/2 719.10 5133.18 16.94
C: Fe 2+ 2p1/2 satellite 725.25 3570.34 11.78
C: Fe 3+ 2p satellite 732.64 760.49 2.51
CPS x 10-3
Binding Energy (eV)

773

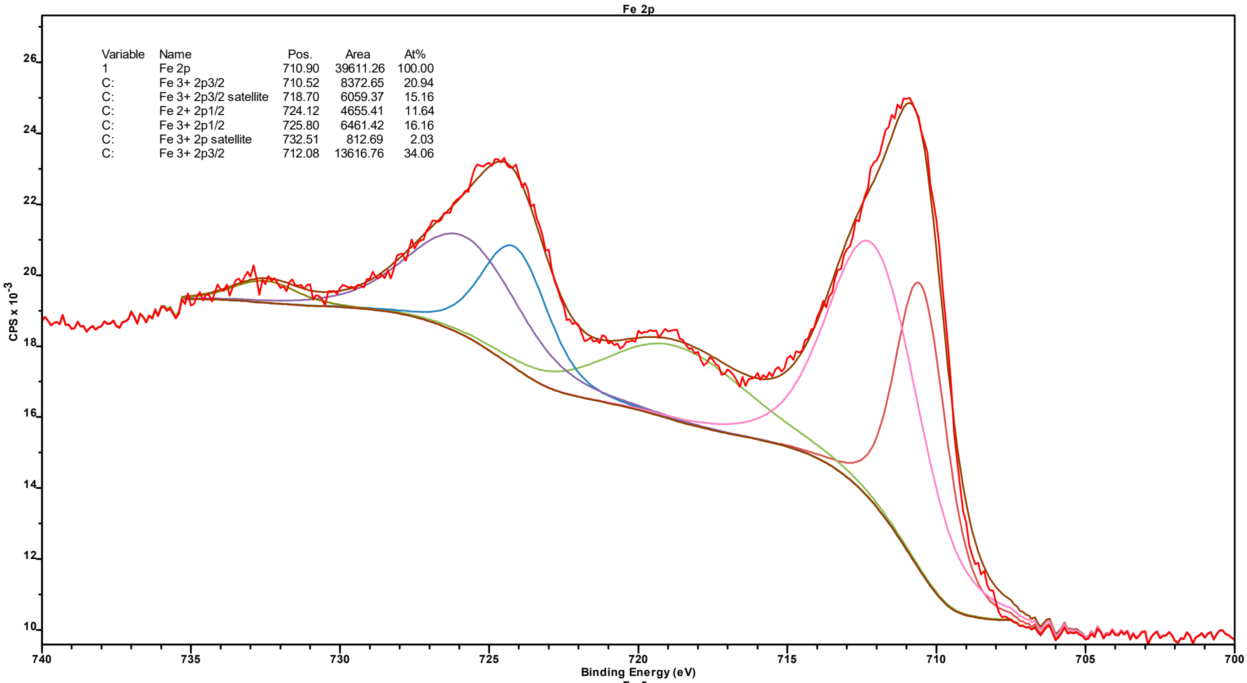
Fe 2p
Variable Name Pos. Area At%
1 Fe 2p 710.90 39611.26 100.00
C: Fe 3+ 2p3/2 710.52 8372.65 20.94
C: Fe 3+ 2p3/2 satellite 718.70 6059.37 15.16
C: Fe 2+ 2p1/2 724.12 4655.41 11.64
C: Fe 3+ 2p1/2 725.80 6461.42 16.16
C: Fe 3+ 2p satellite 732.51 812.69 2.03
C: Fe 3+ 2p3/2 712.08 13616.76 34.06
CPS x 10^-3
Binding Energy (eV)

823

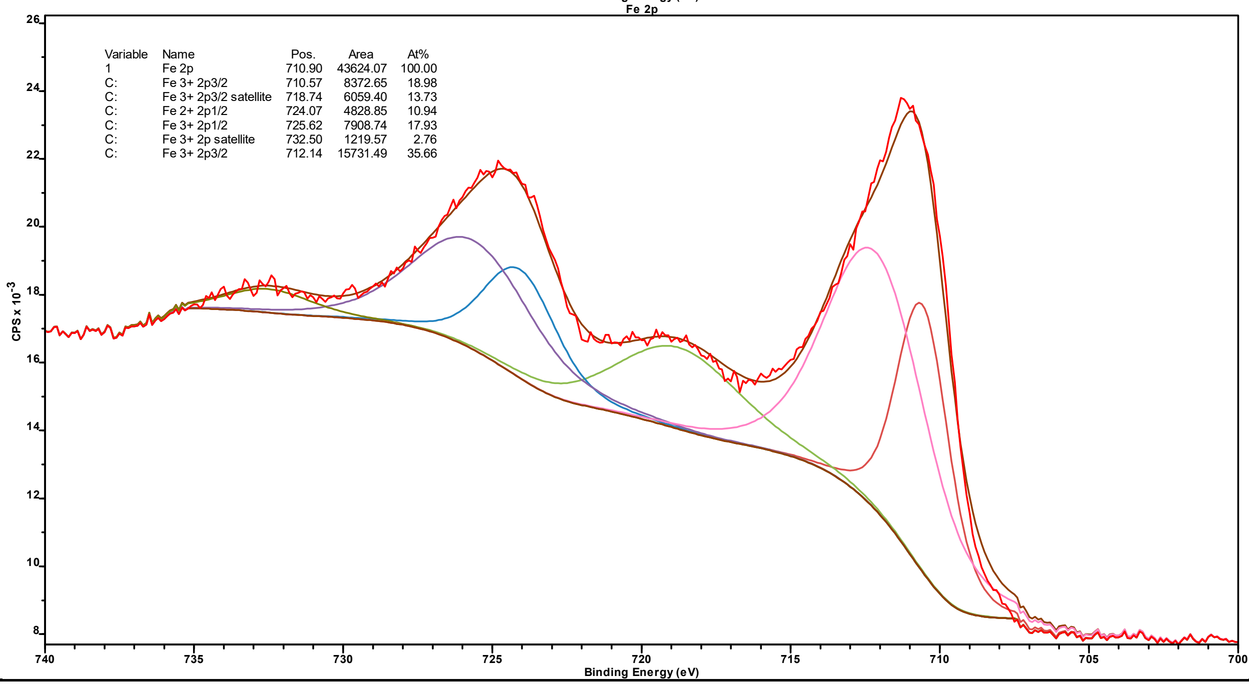
Fe 2p
Variable Name Pos. Area At%
1 Fe 2p 710.90 43624.07 100.00
C: Fe 3+ 2p3/2 710.57 8372.65 18.98
C: Fe 3+ 2p3/2 satellite 718.74 6059.40 13.73
C: Fe 2+ 2p1/2 724.07 4828.85 10.94
C: Fe 3+ 2p1/2 725.62 7908.74 17.93
C: Fe 3+ 2p satellite 732.50 1219.57 2.76
C: Fe 3+ 2p3/2 712.14 15731.49 35.66
CPS x 10^-3
Binding Energy (eV)

873

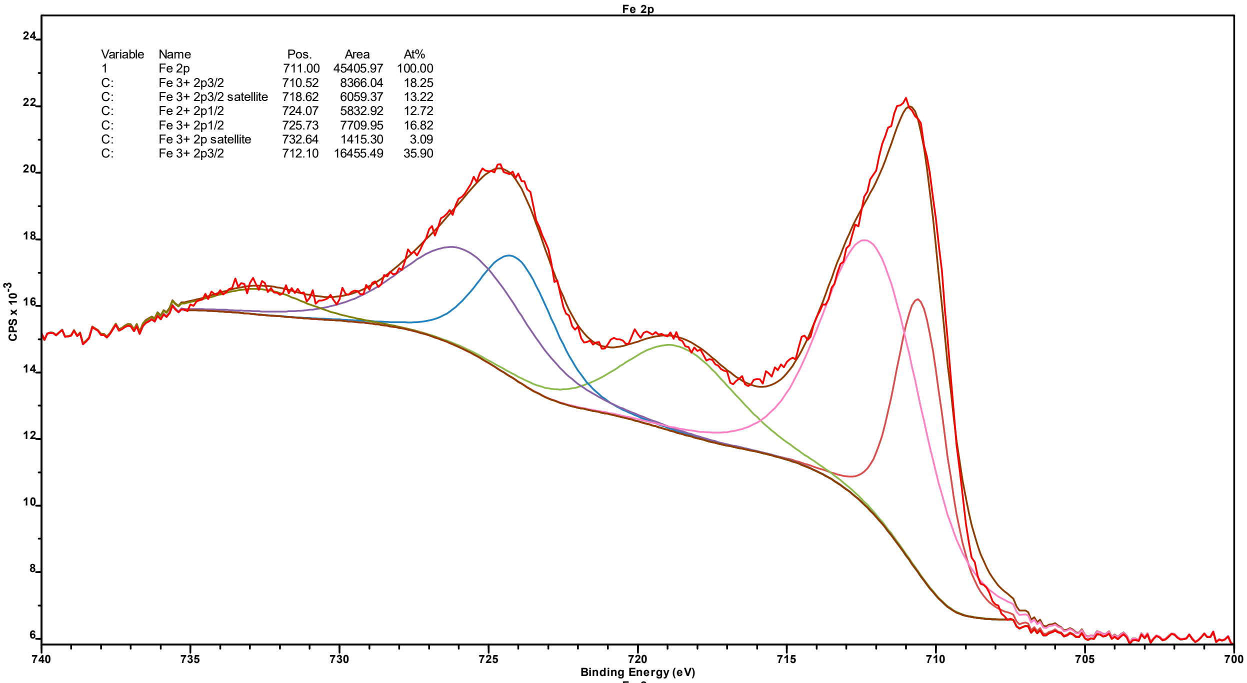
Fe 2p
Variable Name Pos. Area At%
1 Fe 2p 711.00 45405.97 100.00
C: Fe 3+ 2p3/2 710.52 8366.04 18.25
C: Fe 3+ 2p3/2 satellite 718.62 6059.37 13.22
C: Fe 2+ 2p1/2 724.07 5832.92 12.72
C: Fe 3+ 2p1/2 725.73 7709.95 16.82
C: Fe 3+ 2p satellite 732.64 1415.30 3.09
C: Fe 3+ 2p3/2 712.10 16455.49 35.90
CPS x 10-3
Binding Energy (eV)

923

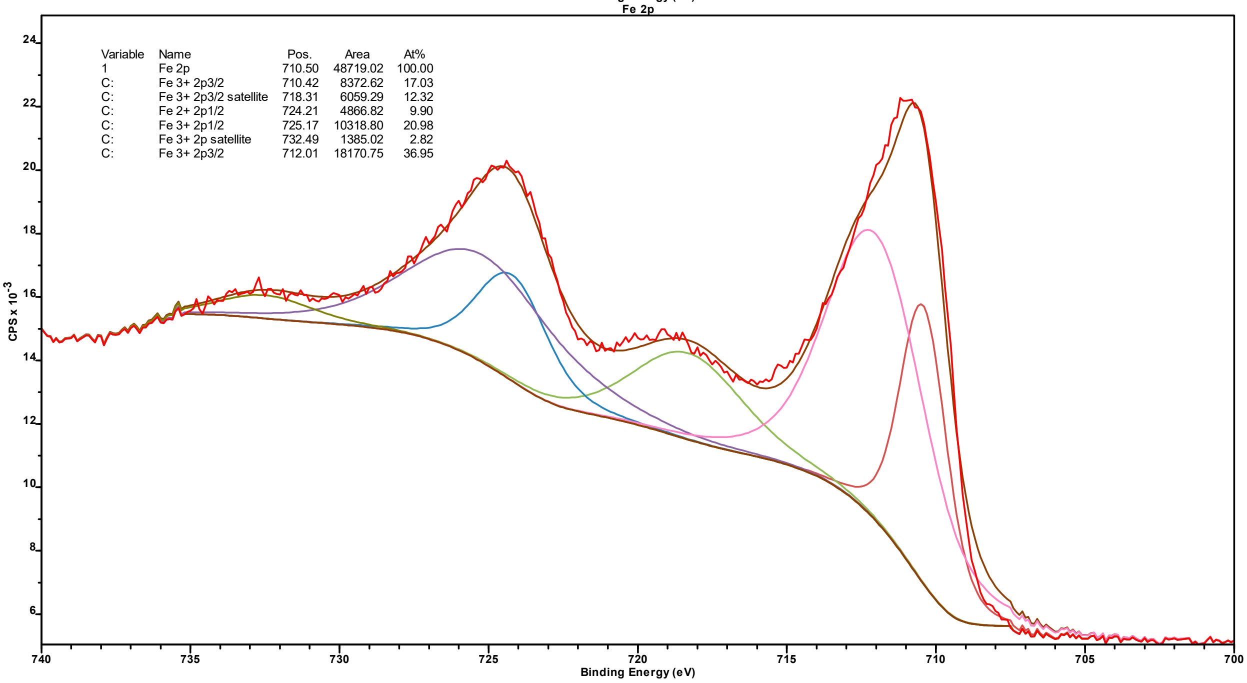
Fe 2p
Variable Name Pos. Area At%
1 Fe 2p 710.50 48719.02 100.00
C: Fe 3+ 2p3/2 710.42 8372.62 17.03
C: Fe 3+ 2p3/2 satellite 718.31 6059.29 12.32
C: Fe 2+ 2p1/2 724.21 4866.82 9.90
C: Fe 3+ 2p1/2 725.17 10318.80 20.98
C: Fe 3+ 2p satellite 732.49 1385.02 2.82
C: Fe 3+ 2p3/2 712.01 18170.75 36.95
CPS x 10-3
Binding Energy (eV)

**T (K)**	**Bi 4f**
300	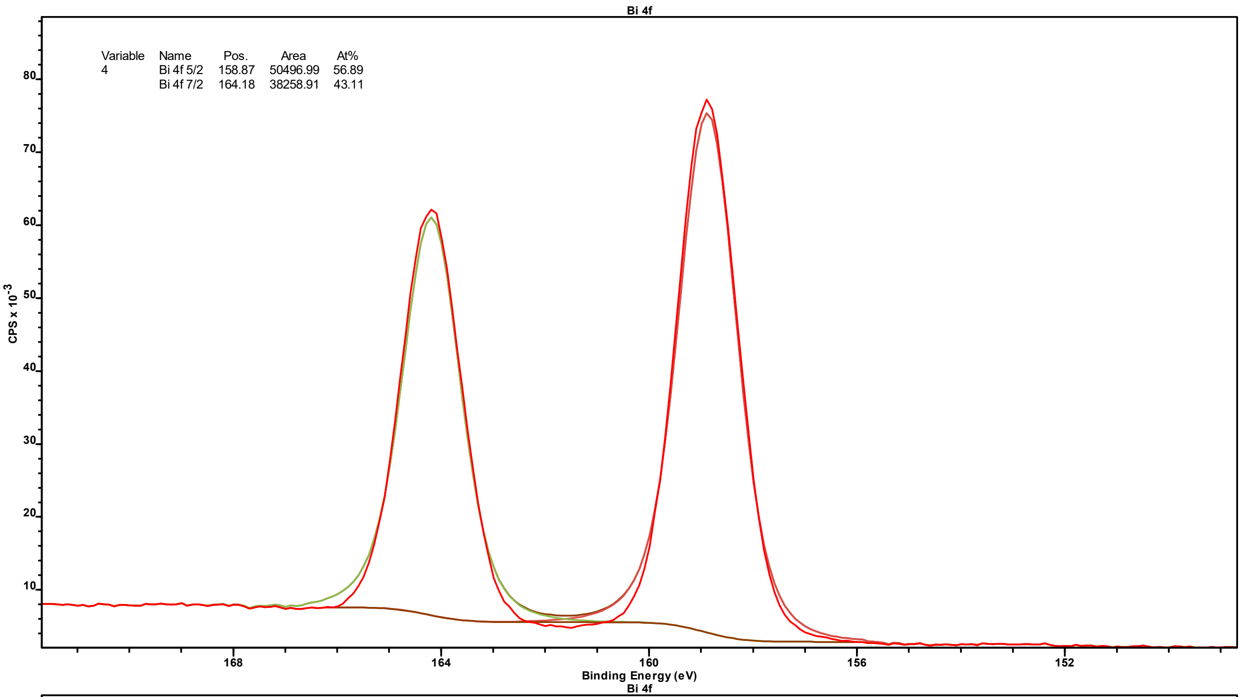
373	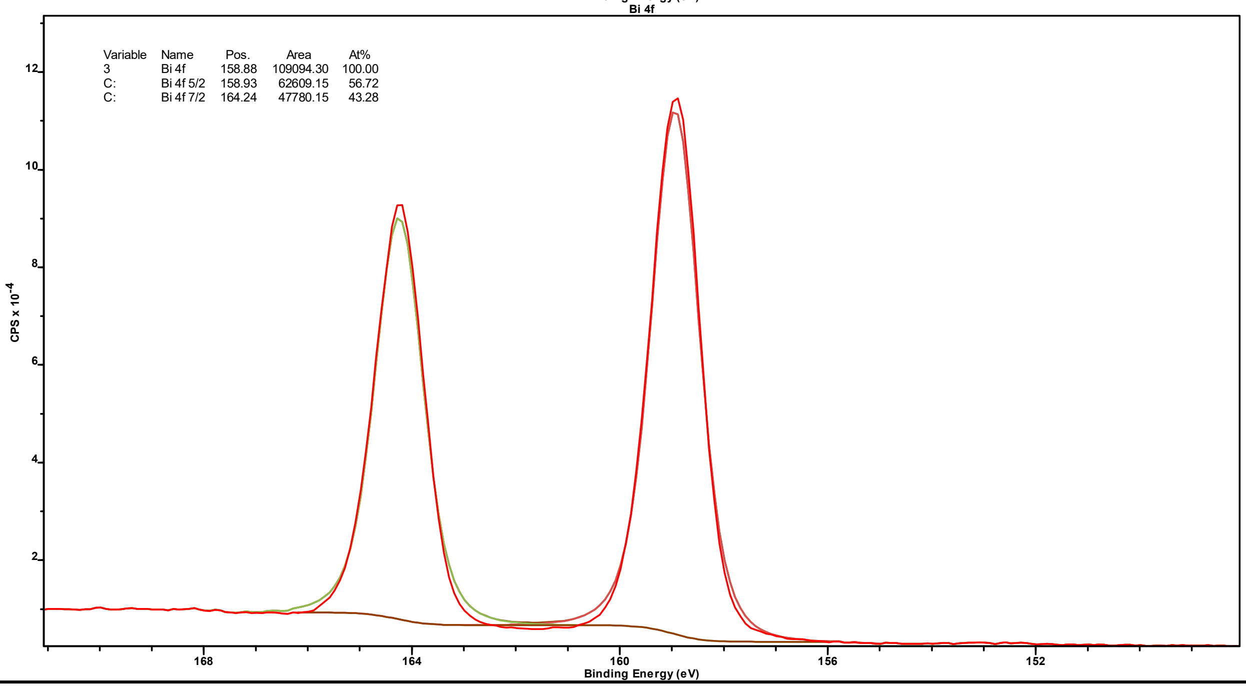

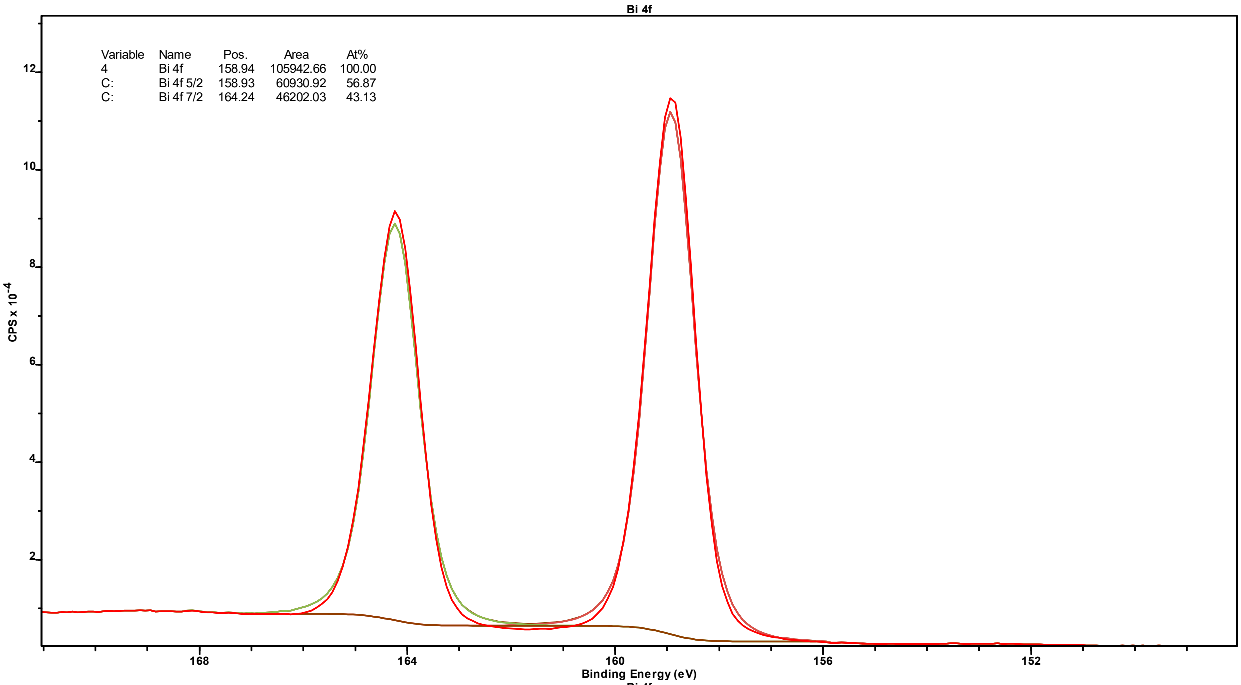
Bi 4f
Variable Name Pos. Area At%
4 Bi 4f 158.94 105942.66 100.00
C: Bi 4f 5/2 158.93 60930.92 56.87
C: Bi 4f 7/2 164.24 46202.03 43.13
CPS x 10^-4
Binding Energy (eV)

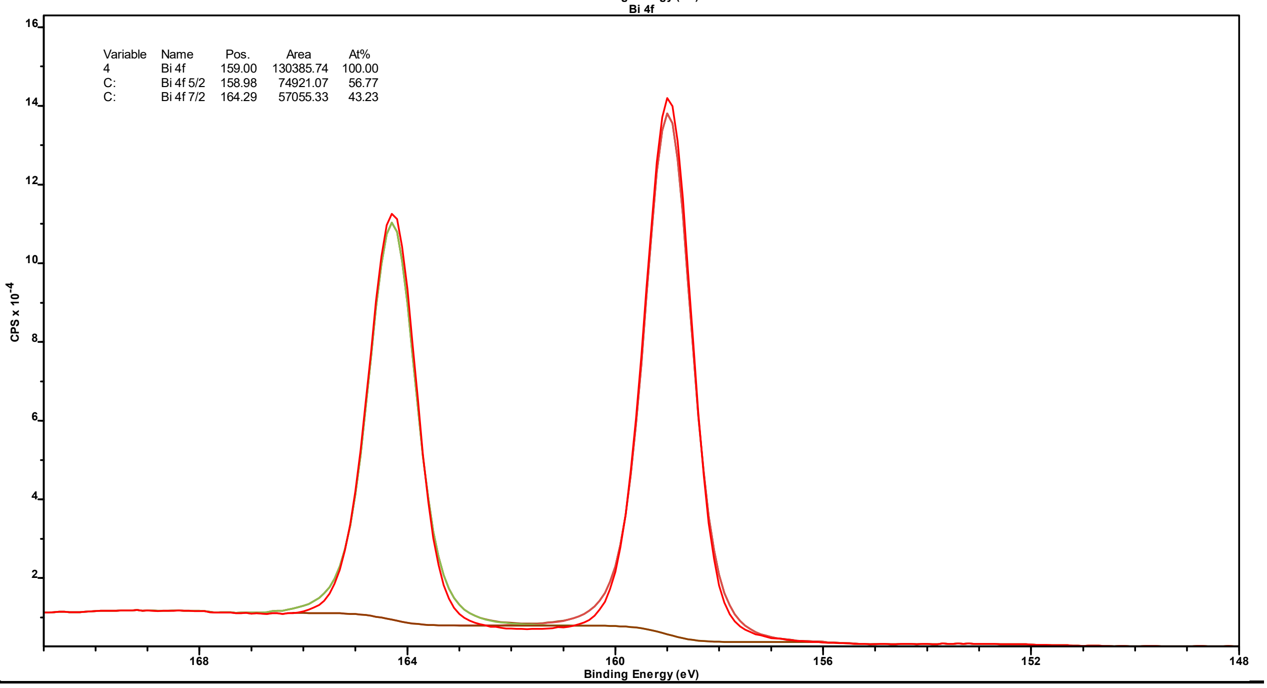
Bi 4f
Variable Name Pos. Area At%
4 Bi 4f 159.00 130385.74 100.00
C: Bi 4f 5/2 158.98 74921.07 56.77
C: Bi 4f 7/2 164.29 57055.33 43.23
CPS x 10^-4
Binding Energy (eV)

573

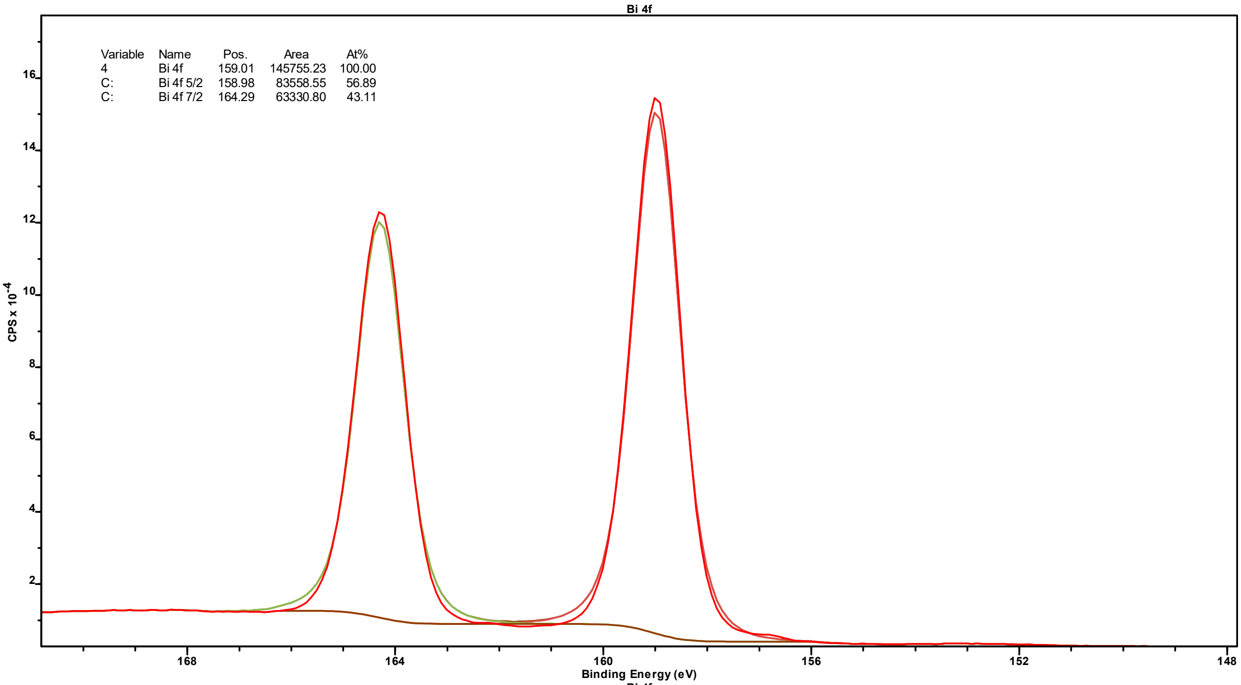
Bi 4f
Variable Name Pos. Area At%
4 Bi 4f 159.01 145755.23 100.00
C: Bi 4f 5/2 158.98 83558.55 56.89
C: Bi 4f 7/2 164.29 63330.80 43.11
CPS x 10^-4
Binding Energy (eV)

623

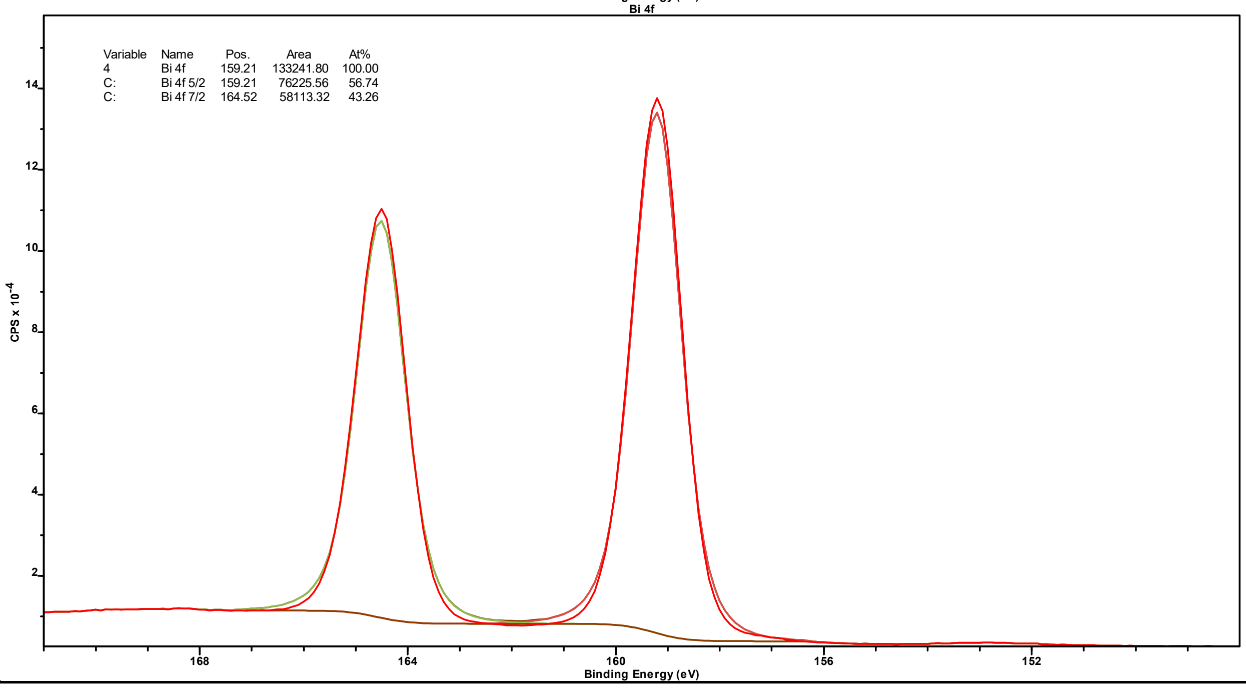
Bi 4f
Variable Name Pos. Area At%
4 Bi 4f 159.21 133241.80 100.00
C: Bi 4f 5/2 159.21 76225.56 56.74
C: Bi 4f 7/2 164.52 58113.32 43.26
CPS x 10^-4
Binding Energy (eV)

673

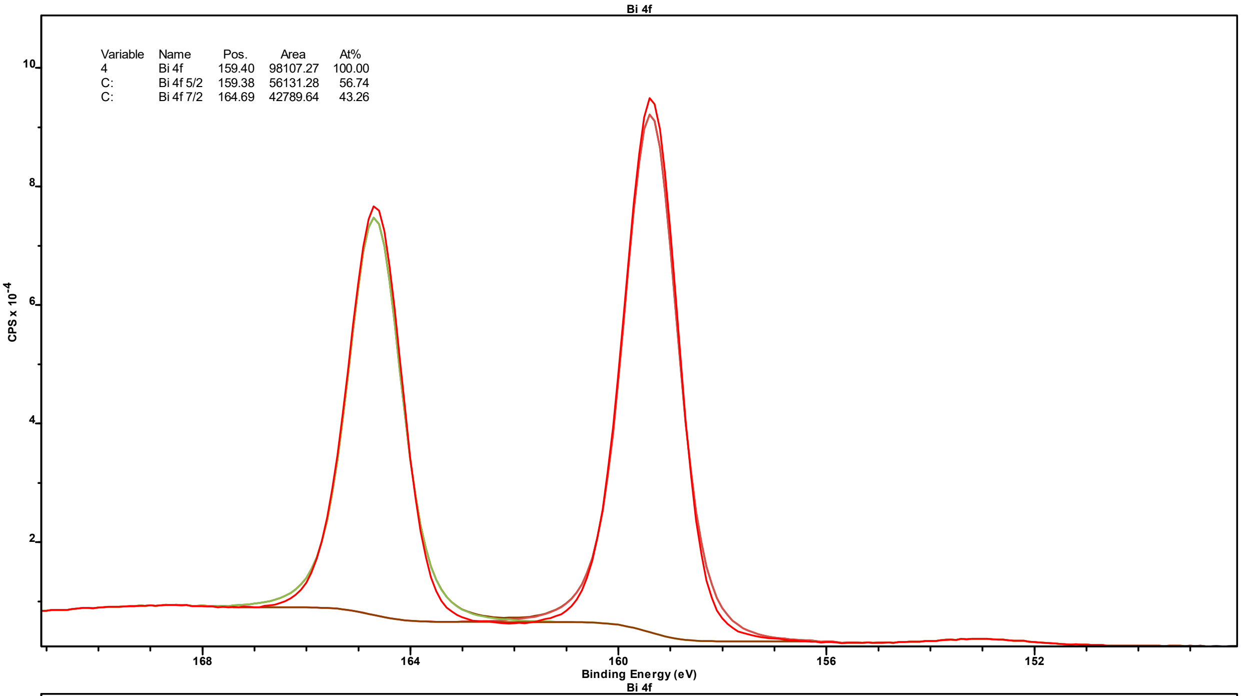
Bi 4f
Variable Name Pos. Area At%
4 Bi 4f 159.40 98107.27 100.00
C: Bi 4f 5/2 159.38 56131.28 56.74
C: Bi 4f 7/2 164.69 42789.64 43.26
CPS x 10^-4
Binding Energy (eV)

723

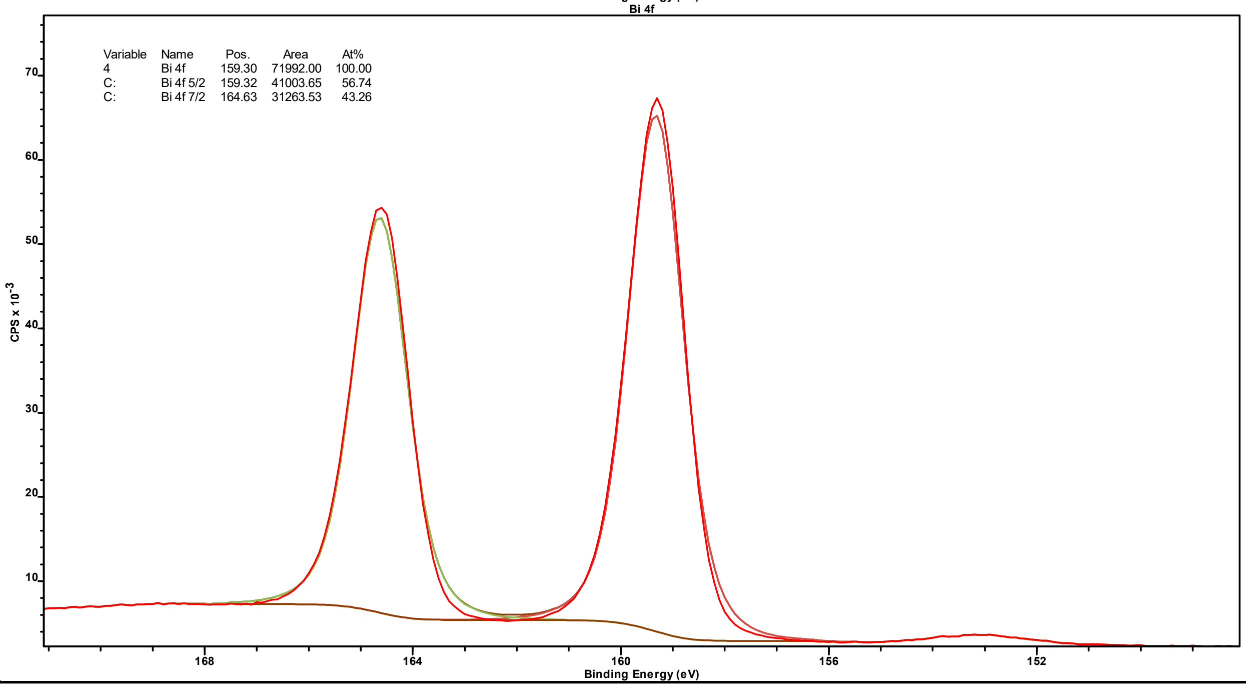
Bi 4f
Variable Name Pos. Area At%
4 Bi 4f 159.30 71992.00 100.00
C: Bi 4f 5/2 159.32 41003.65 56.74
C: Bi 4f 7/2 164.63 31263.53 43.26
CPS x 10^-3
Binding Energy (eV)

773

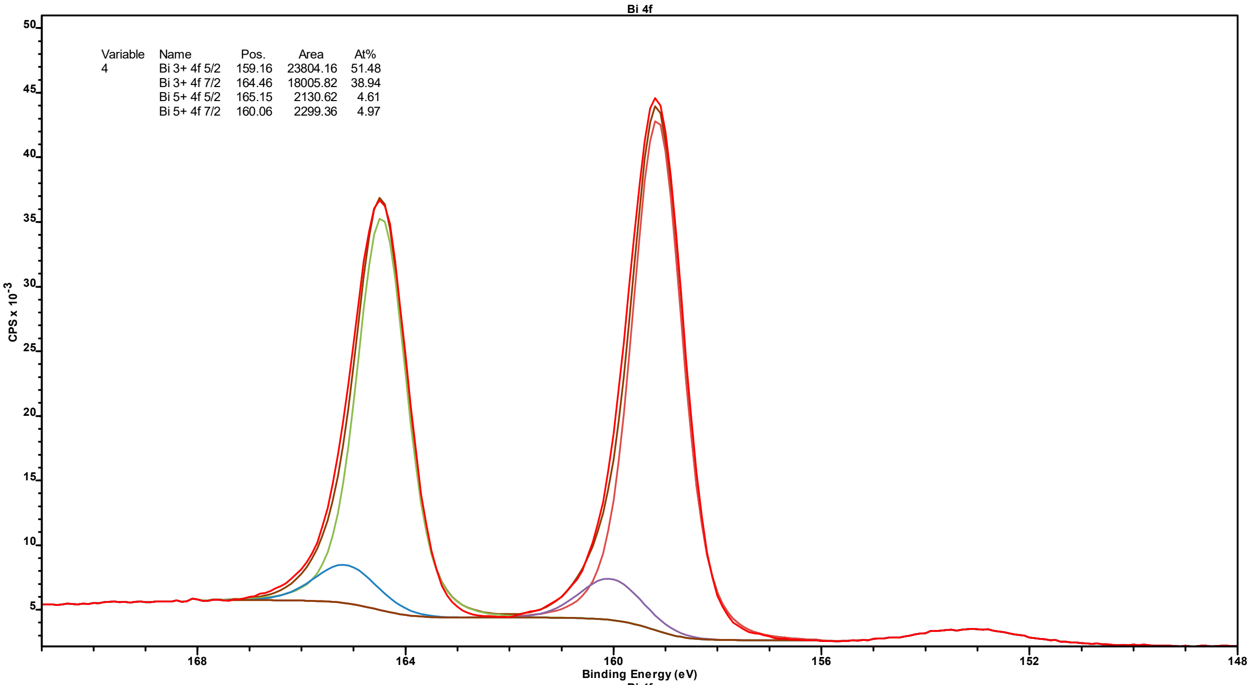
Bi 4f
Variable Name Pos. Area At%
4 Bi 3+ 4f 5/2 159.16 23804.16 51.48
Bi 3+ 4f 7/2 164.46 18005.82 38.94
Bi 5+ 4f 5/2 165.15 2130.62 4.61
Bi 5+ 4f 7/2 160.06 2299.36 4.97
CPS x 10^-3
50
45
40
35
30
25
20
15
10
5
168
164
160
156
152
148
Binding Energy (eV)

823

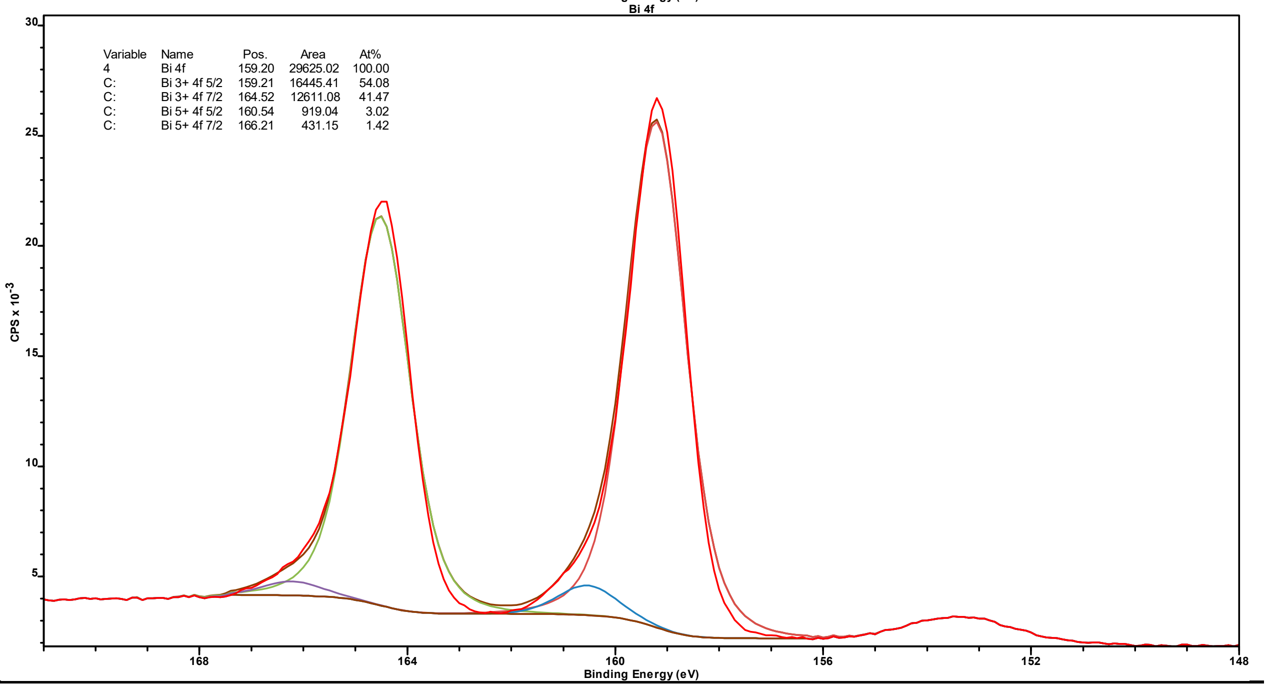
Bi 4f
Variable Name Pos. Area At%
4 Bi 4f 159.20 29625.02 100.00
C: Bi 3+ 4f 5/2 159.21 16445.41 54.08
C: Bi 3+ 4f 7/2 164.52 12611.08 41.47
C: Bi 5+ 4f 5/2 160.54 919.04 3.02
C: Bi 5+ 4f 7/2 166.21 431.15 1.42
CPS x 10^-3
30
25
20
15
10
5
168
164
160
156
152
148
Binding Energy (eV)

873

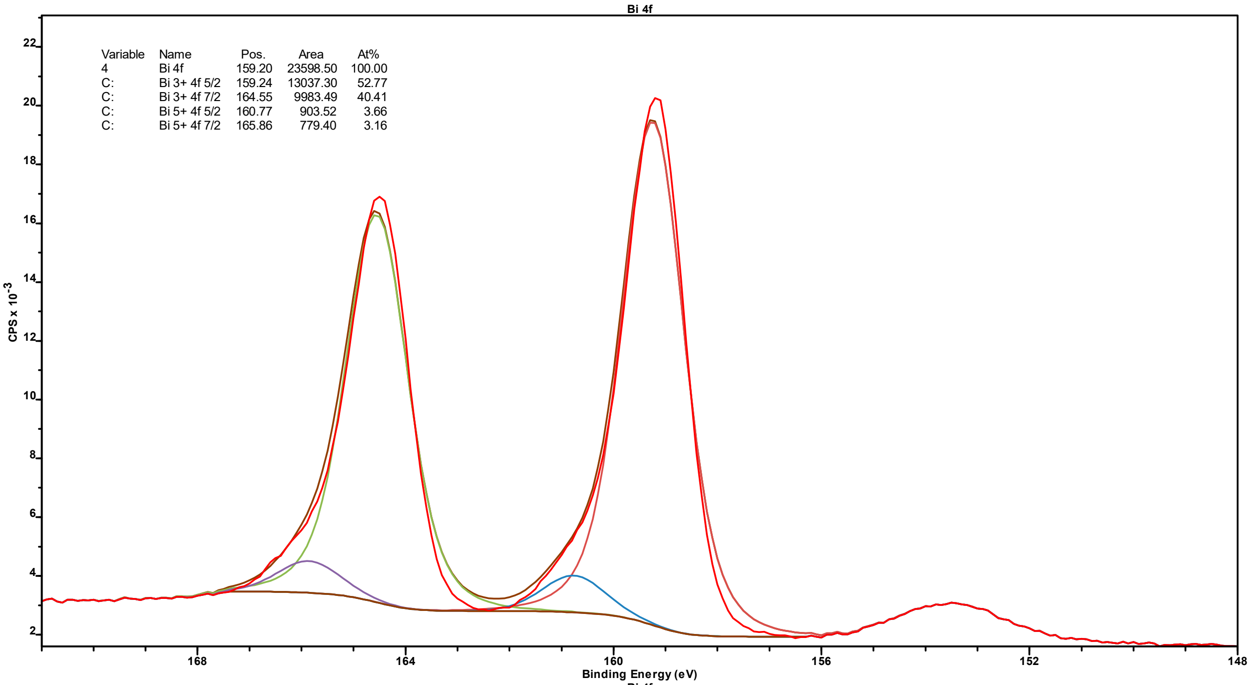
Bi 4f
Variable Name Pos. Area At%
4 Bi 4f 159.20 23598.50 100.00
C: Bi 3+ 4f 5/2 159.24 13037.30 52.77
C: Bi 3+ 4f 7/2 164.55 9983.49 40.41
C: Bi 5+ 4f 5/2 160.77 903.52 3.66
C: Bi 5+ 4f 7/2 165.86 779.40 3.16
CPS x 10^{-3}
Binding Energy (eV)

923

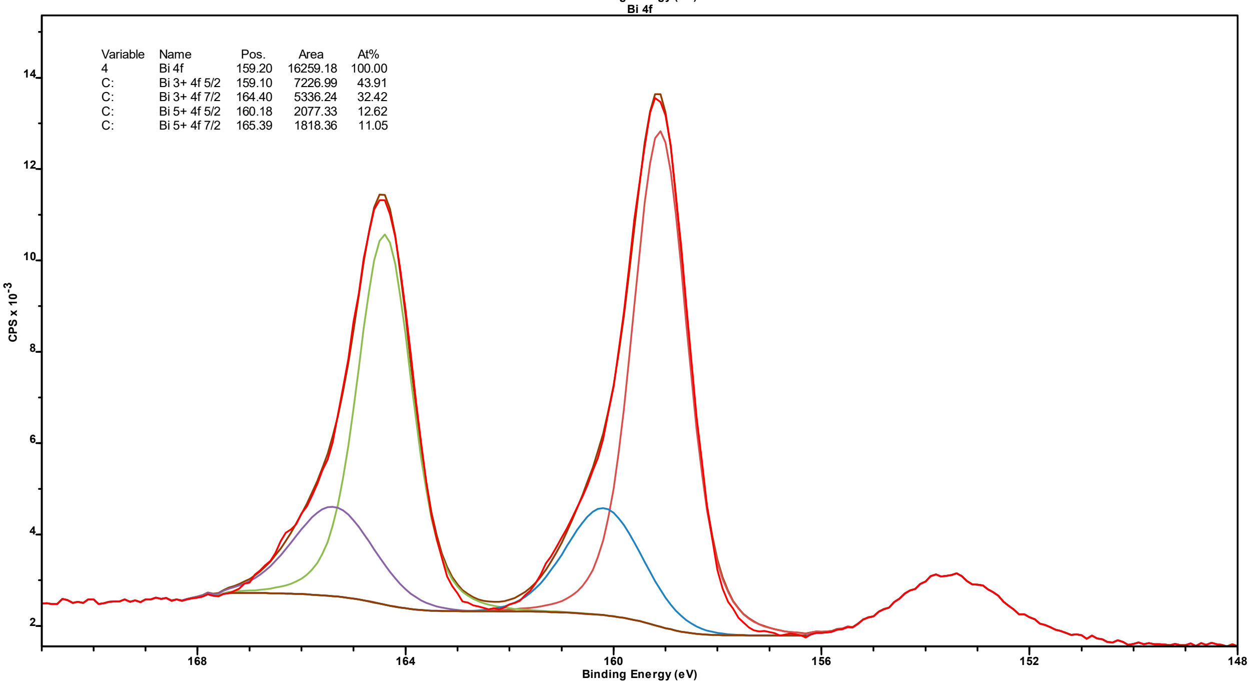
Bi 4f
Variable Name Pos. Area At%
4 Bi 4f 159.20 16259.18 100.00
C: Bi 3+ 4f 5/2 159.10 7226.99 43.91
C: Bi 3+ 4f 7/2 164.40 5336.24 32.42
C: Bi 5+ 4f 5/2 160.18 2077.33 12.62
C: Bi 5+ 4f 7/2 165.39 1818.36 11.05
CPS x 10^{-3}
Binding Energy (eV)

**T (K)**	**O 1s**
300	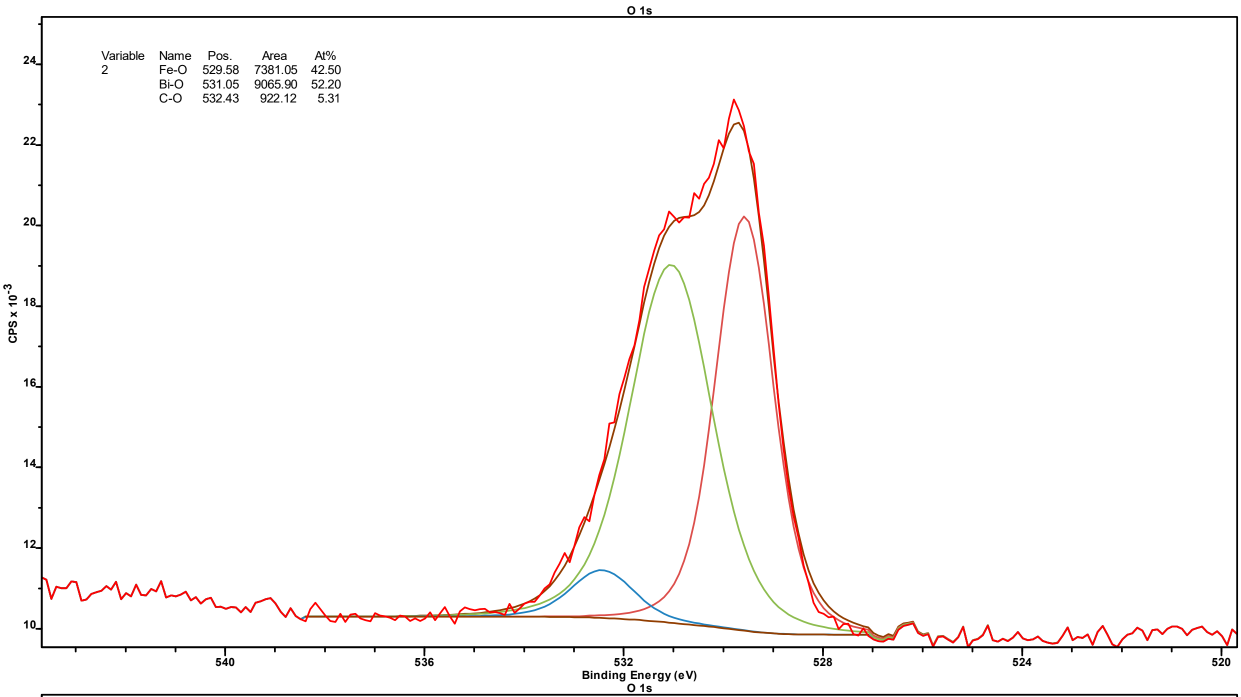
373	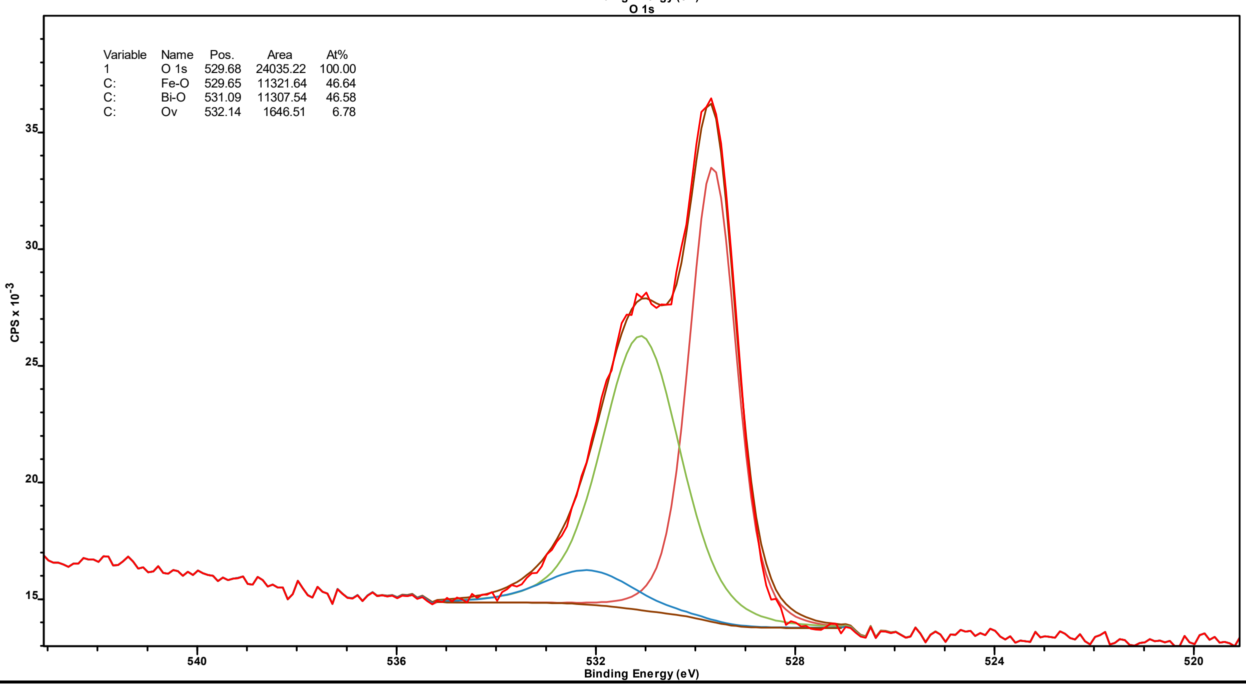

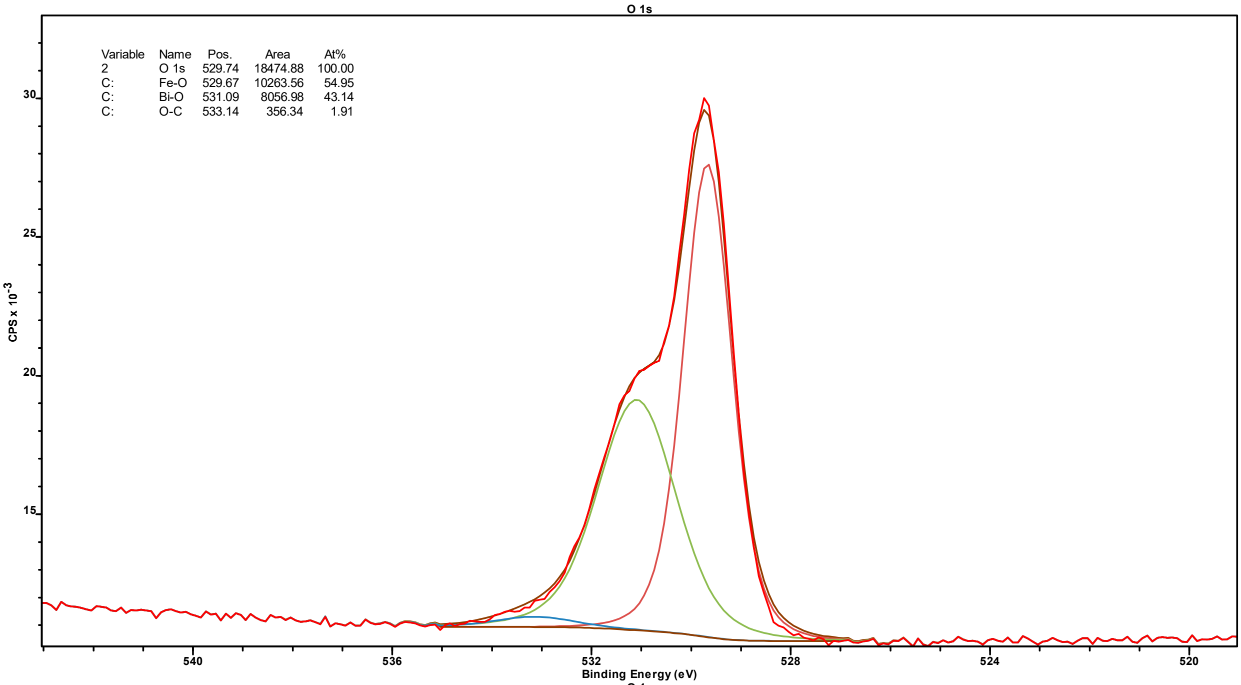
O 1s
Variable Name Pos. Area At%
2 O 1s 529.74 18474.88 100.00
C: Fe-O 529.67 10263.56 54.95
C: Bi-O 531.09 8056.98 43.14
C: O-C 533.14 356.34 1.91
CPS x 10-3
30
25
20
15
540
536
532
528
524
520
Binding Energy (eV)

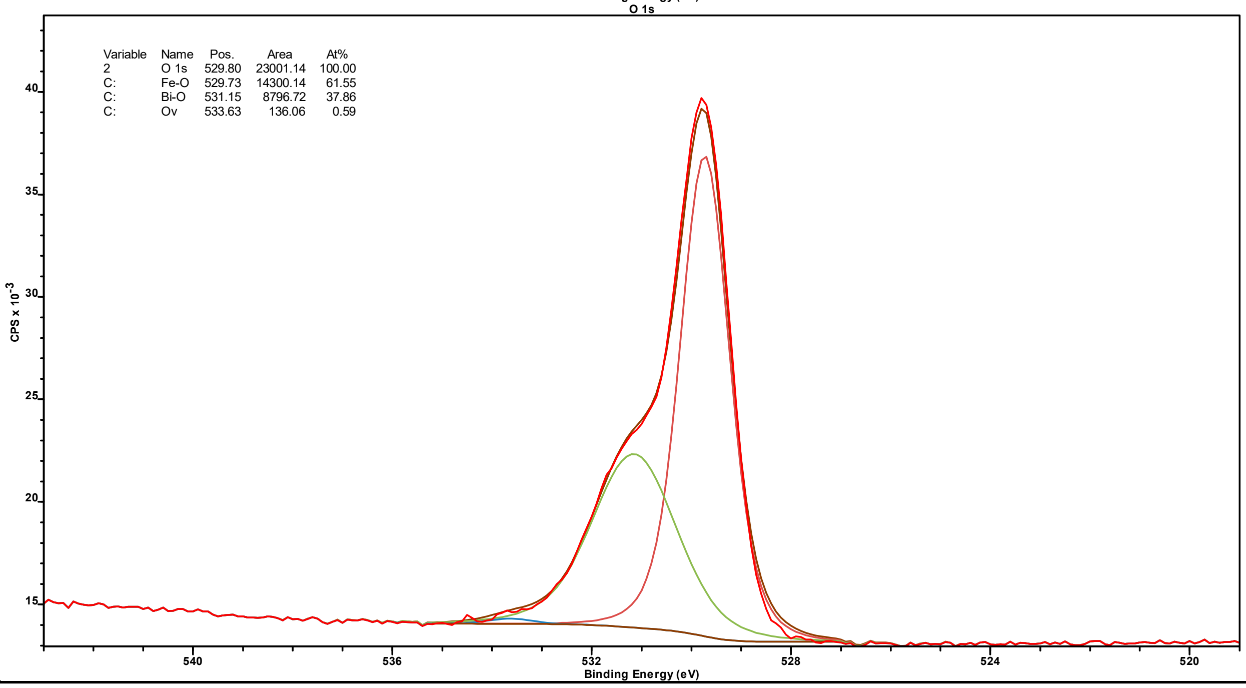
O 1s
Variable Name Pos. Area At%
2 O 1s 529.80 23001.14 100.00
C: Fe-O 529.73 14300.14 61.55
C: Bi-O 531.15 8796.72 37.86
C: Ov 533.63 136.06 0.59
CPS x 10-3
40
35
30
25
20
15
540
536
532
528
524
520
Binding Energy (eV)

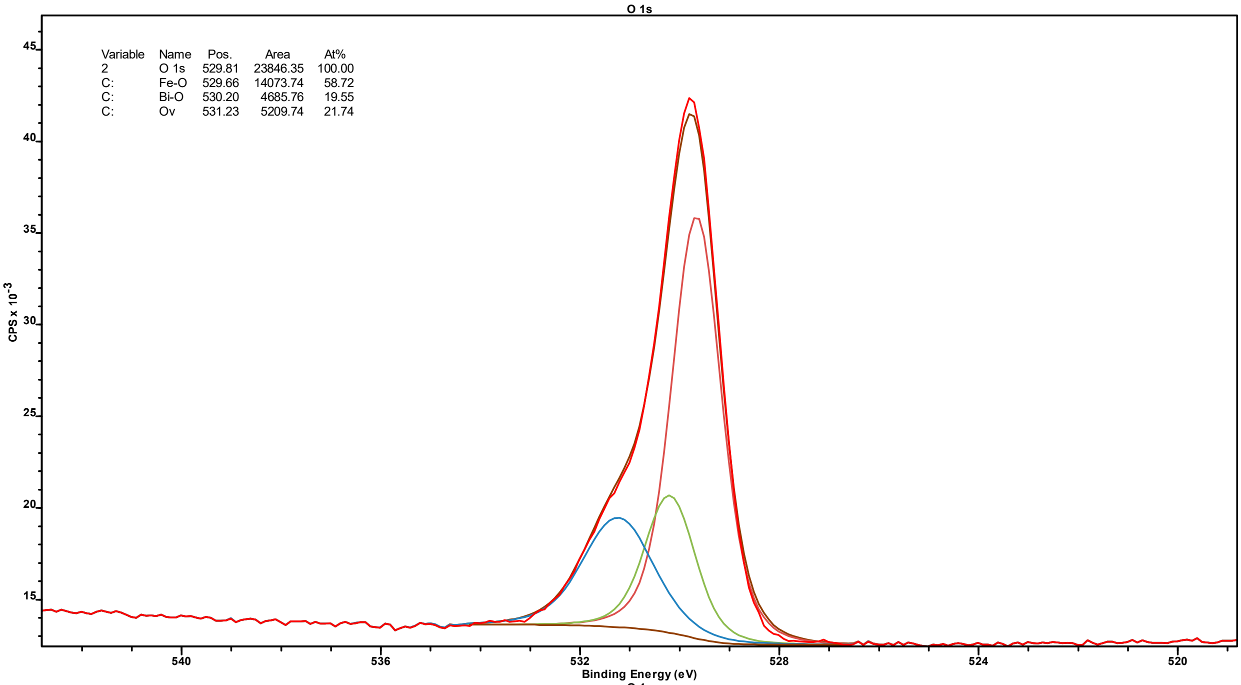
O 1s
Variable Name Pos. Area At%
2 O 1s 529.81 23846.35 100.00
C: Fe-O 529.66 14073.74 58.72
C: Bi-O 530.20 4685.76 19.55
C: Ov 531.23 5209.74 21.74
CPS x 10⁻³
Binding Energy (eV)

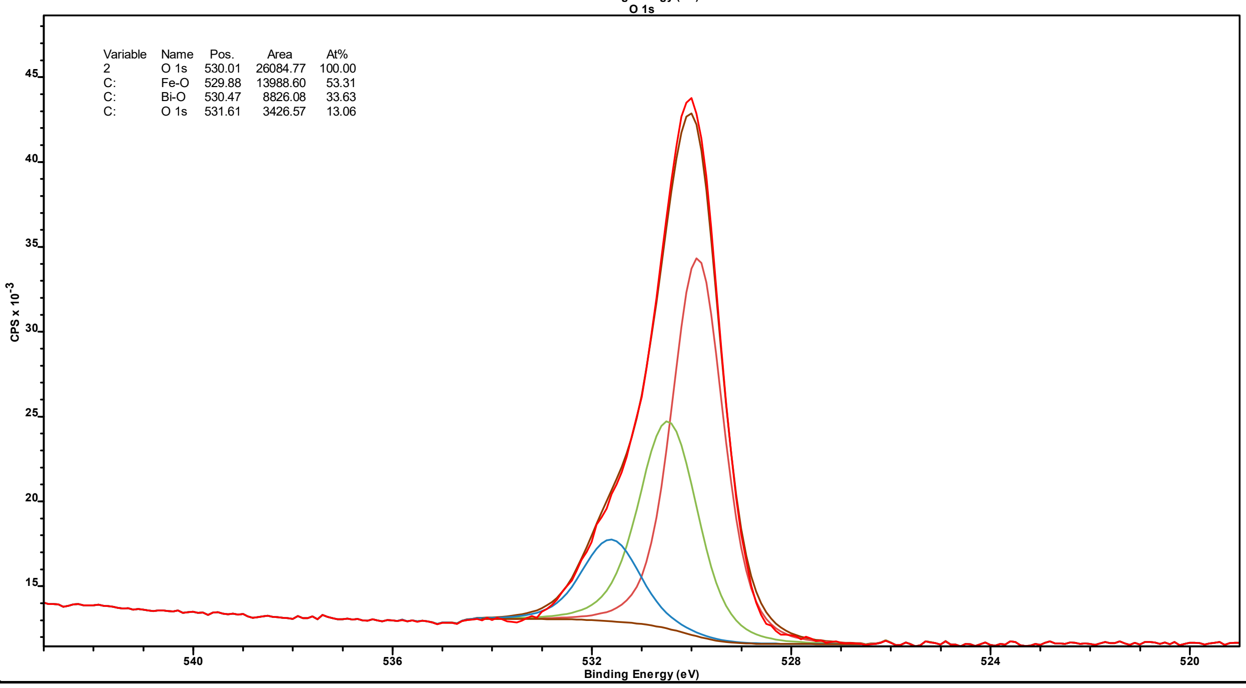
O 1s
Variable Name Pos. Area At%
2 O 1s 530.01 26084.77 100.00
C: Fe-O 529.88 13988.60 53.31
C: Bi-O 530.47 8826.08 33.63
C: O 1s 531.61 3426.57 13.06
CPS x 10⁻³
Binding Energy (eV)

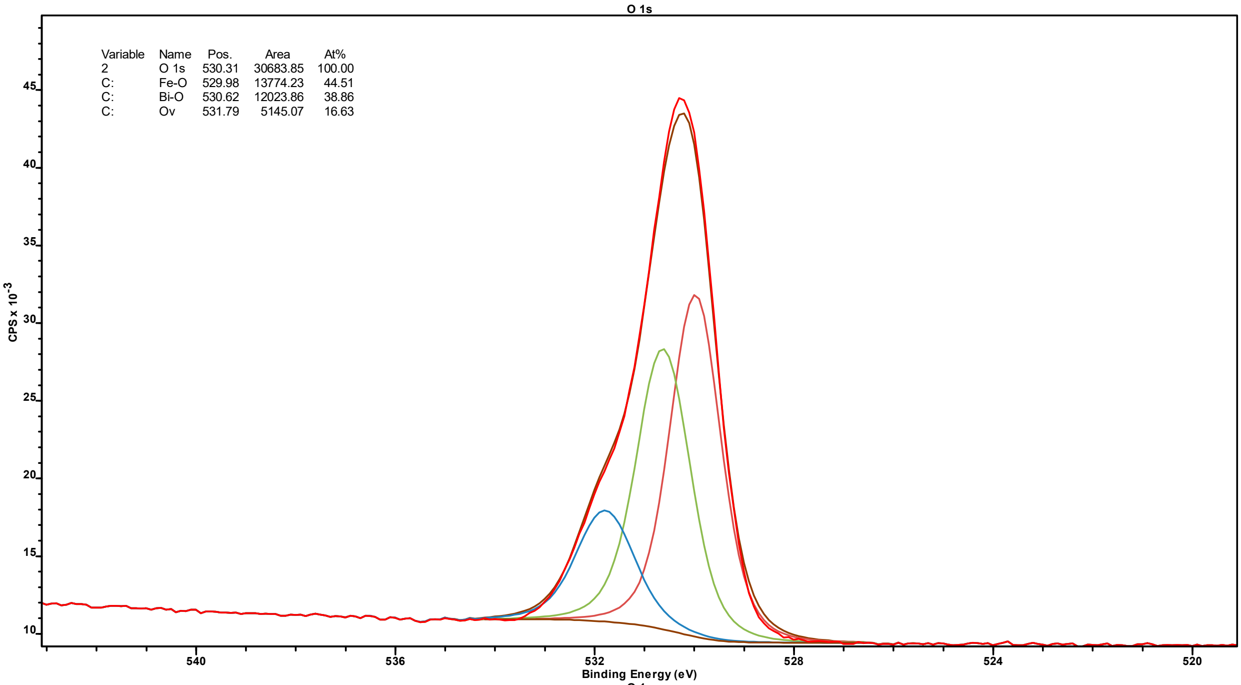
O 1s
Variable Name Pos. Area At%
2 O 1s 530.31 30683.85 100.00
C: Fe-O 529.98 13774.23 44.51
C: Bi-O 530.62 12023.86 38.86
C: Ov 531.79 5145.07 16.63
CPS x 10-3
45
40
35
30
25
20
15
10
540
536
532
528
524
520
Binding Energy (eV)

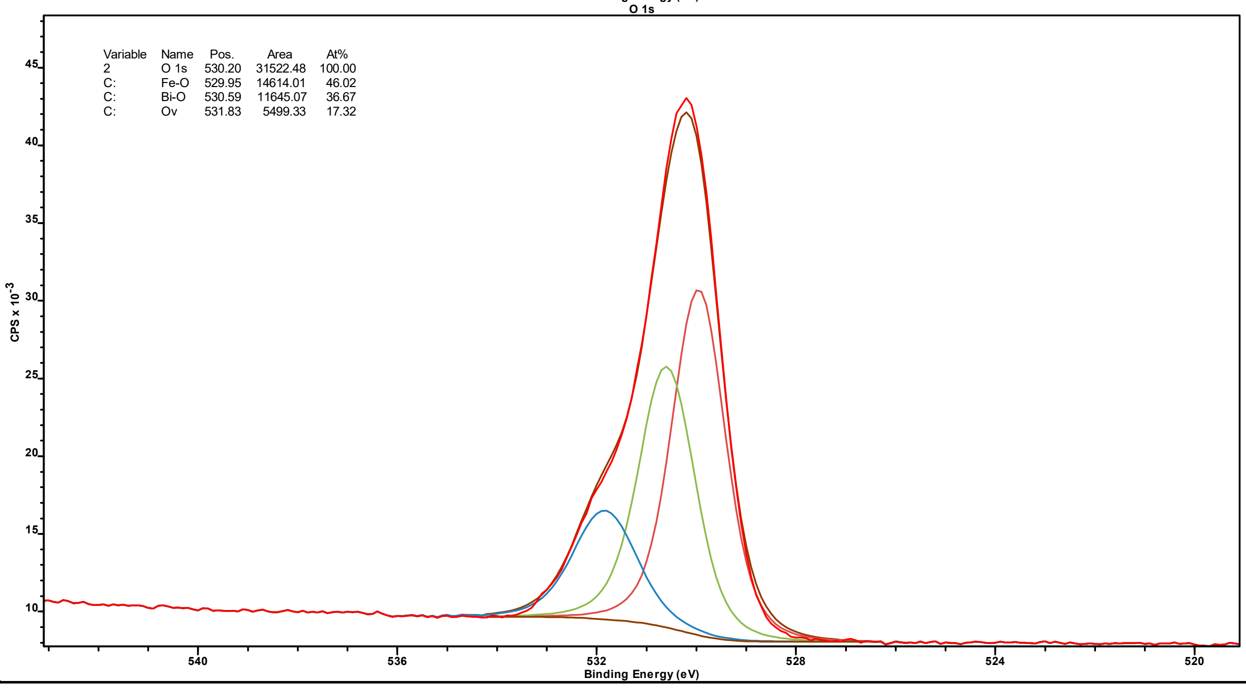
O 1s
Variable Name Pos. Area At%
2 O 1s 530.20 31522.48 100.00
C: Fe-O 529.95 14614.01 46.02
C: Bi-O 530.59 11645.07 36.67
C: Ov 531.83 5499.33 17.32
CPS x 10-3
45
40
35
30
25
20
15
10
540
536
532
528
524
520
Binding Energy (eV)

773

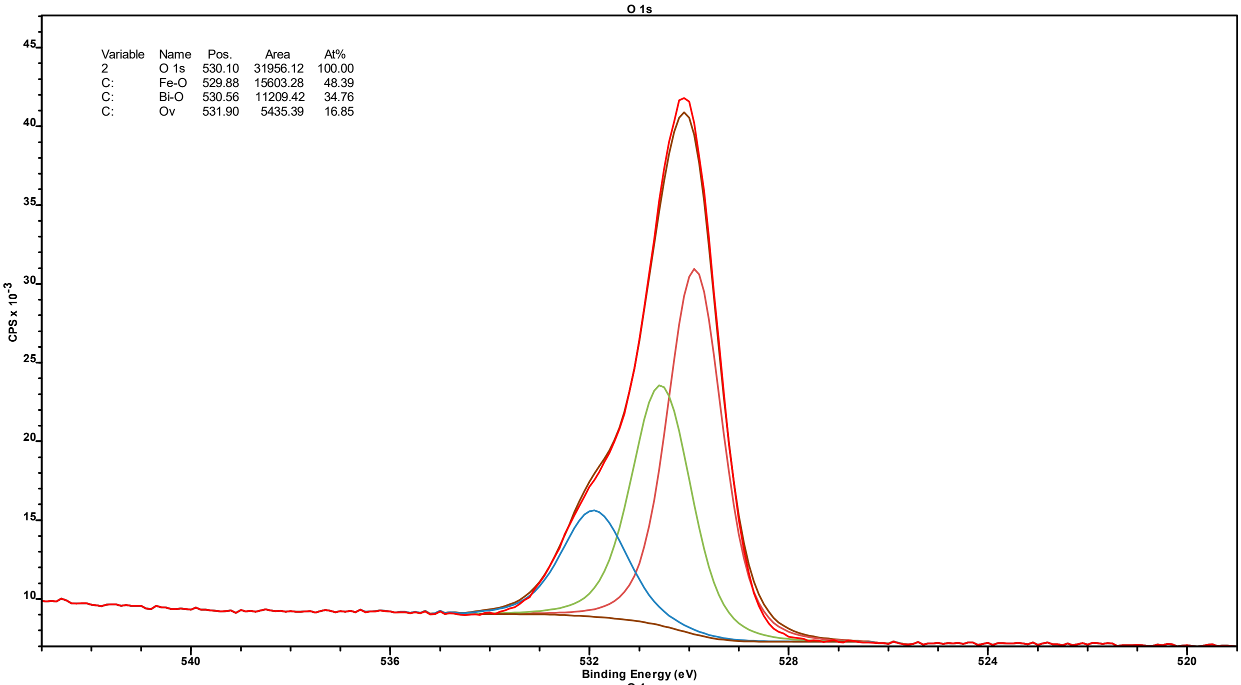
O 1s
Variable Name Pos. Area At%
2 O 1s 530.10 31956.12 100.00
C: Fe-O 529.88 15603.28 48.39
C: Bi-O 530.56 11209.42 34.76
C: Ov 531.90 5435.39 16.85
CPS x 10^{-3}
Binding Energy (eV)

823

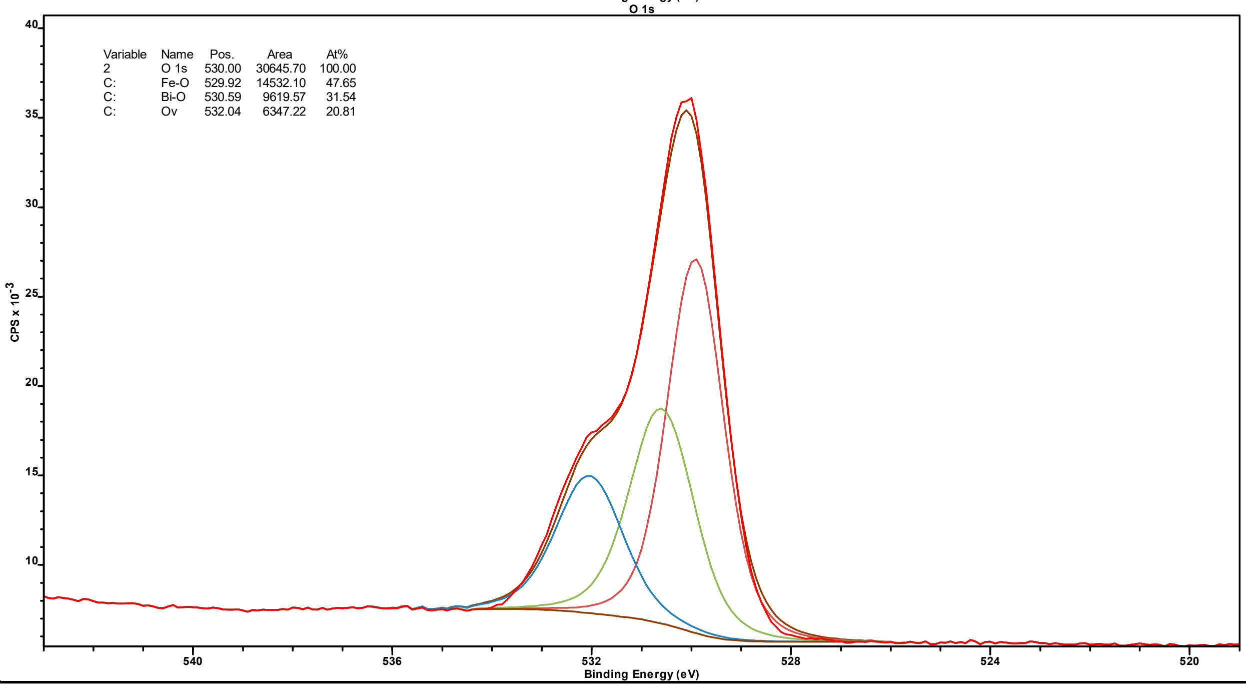
O 1s
Variable Name Pos. Area At%
2 O 1s 530.00 30645.70 100.00
C: Fe-O 529.92 14532.10 47.65
C: Bi-O 530.59 9619.57 31.54
C: Ov 532.04 6347.22 20.81
CPS x 10^{-3}
Binding Energy (eV)

873

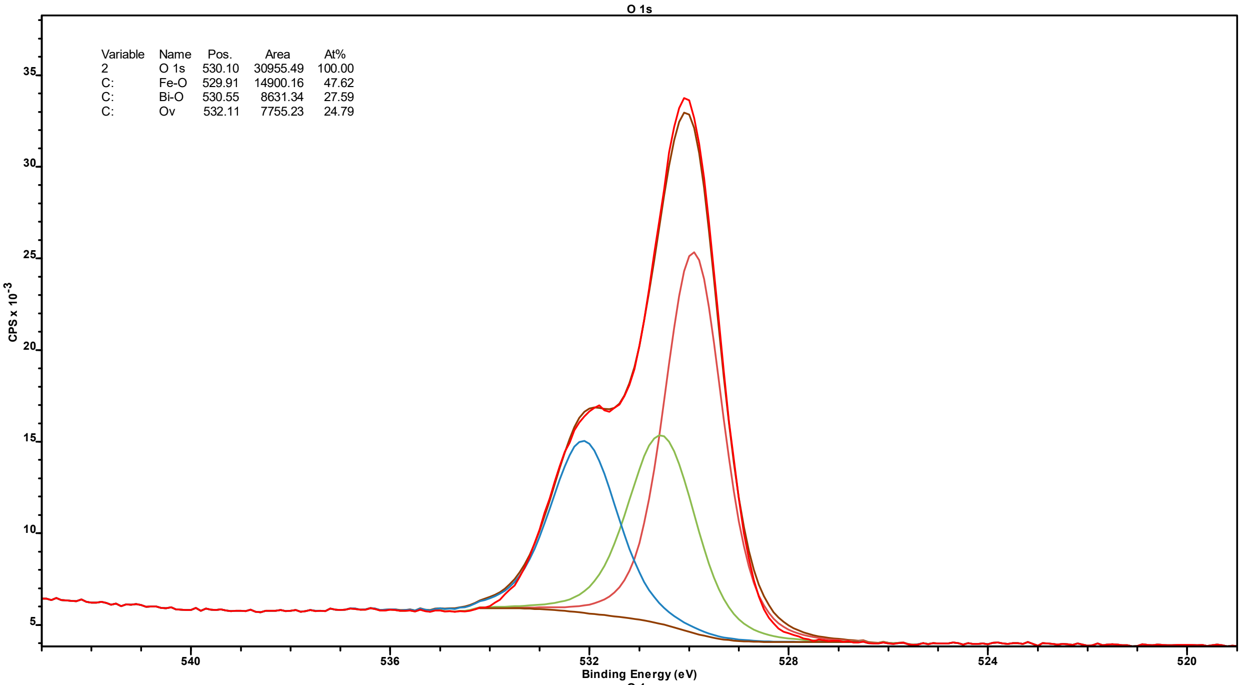
O 1s
Variable Name Pos. Area At%
2 O 1s 530.10 30955.49 100.00
C: Fe-O 529.91 14900.16 47.62
C: Bi-O 530.55 8631.34 27.59
C: Ov 532.11 7755.23 24.79
CPS x 10-3
Binding Energy (eV)

923

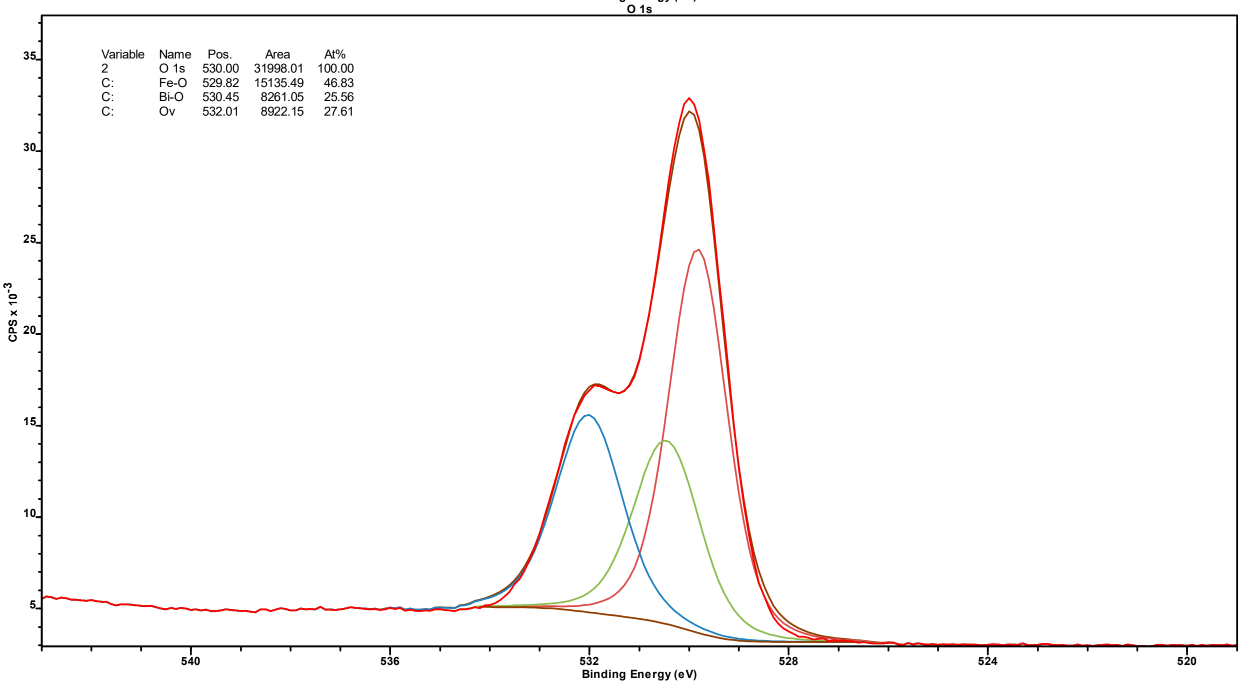
O 1s
Variable Name Pos. Area At%
2 O 1s 530.00 31998.01 100.00
C: Fe-O 529.82 15135.49 46.83
C: Bi-O 530.45 8261.05 25.56
C: Ov 532.01 8922.15 27.61
CPS x 10-3
Binding Energy (eV)

**T (K)**	**C 1s**
300	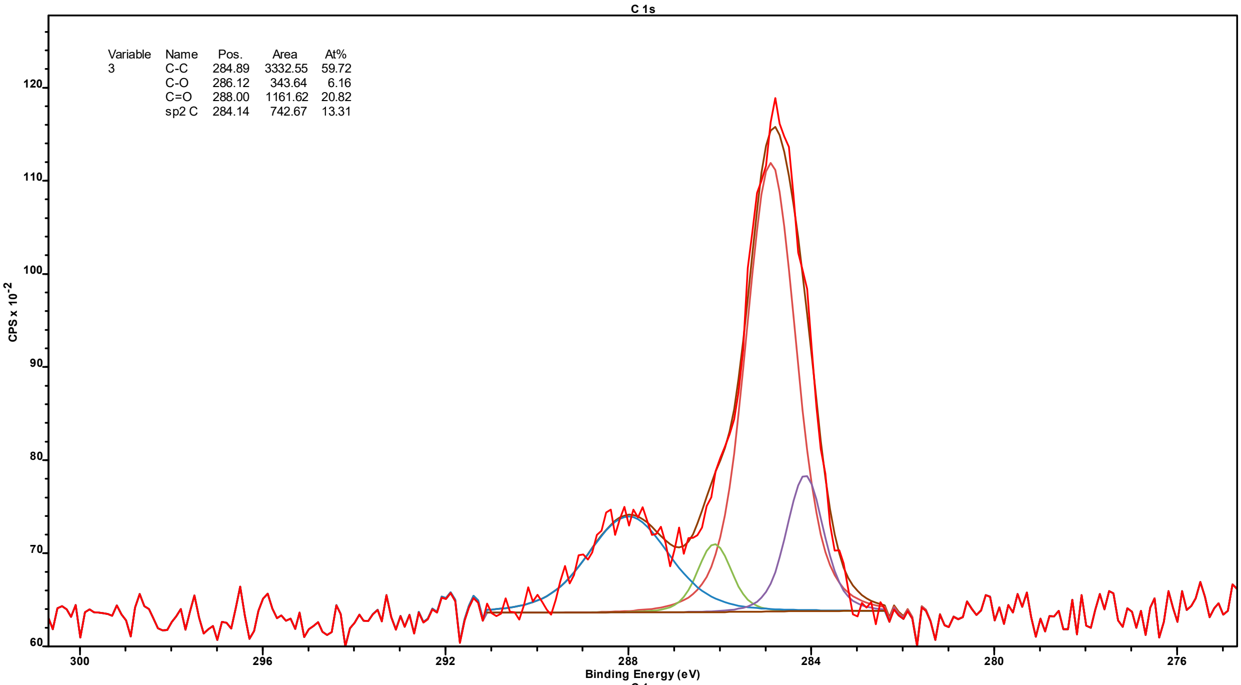
373	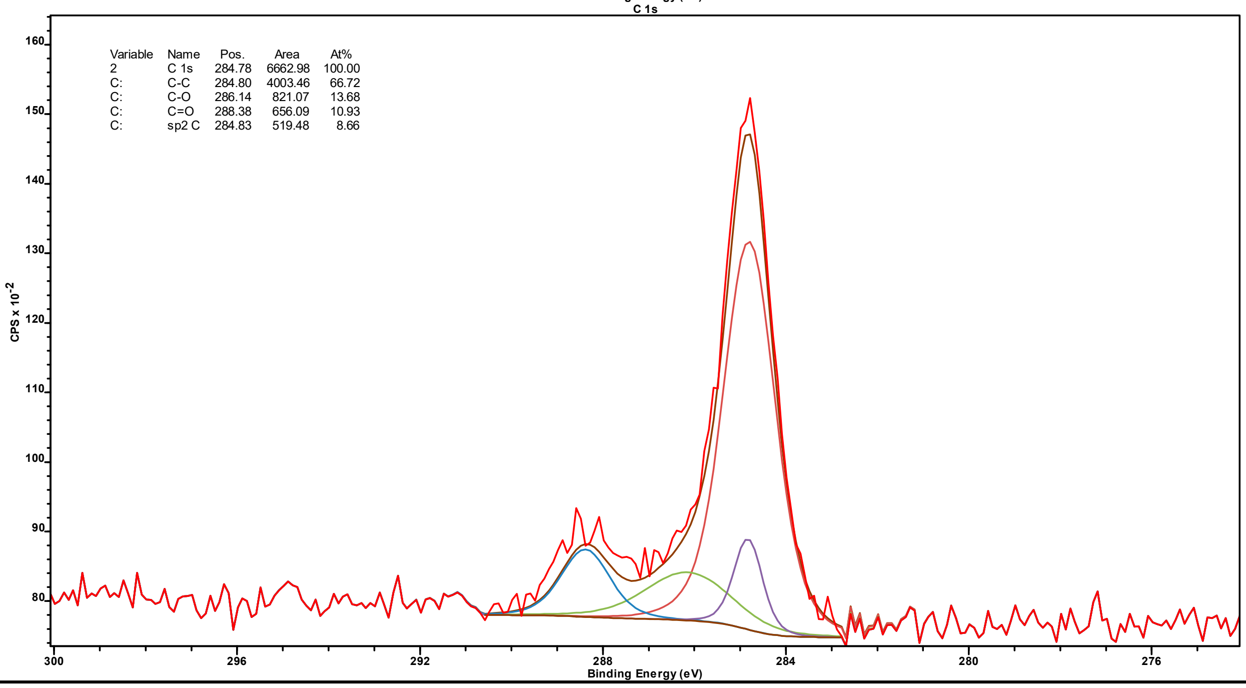

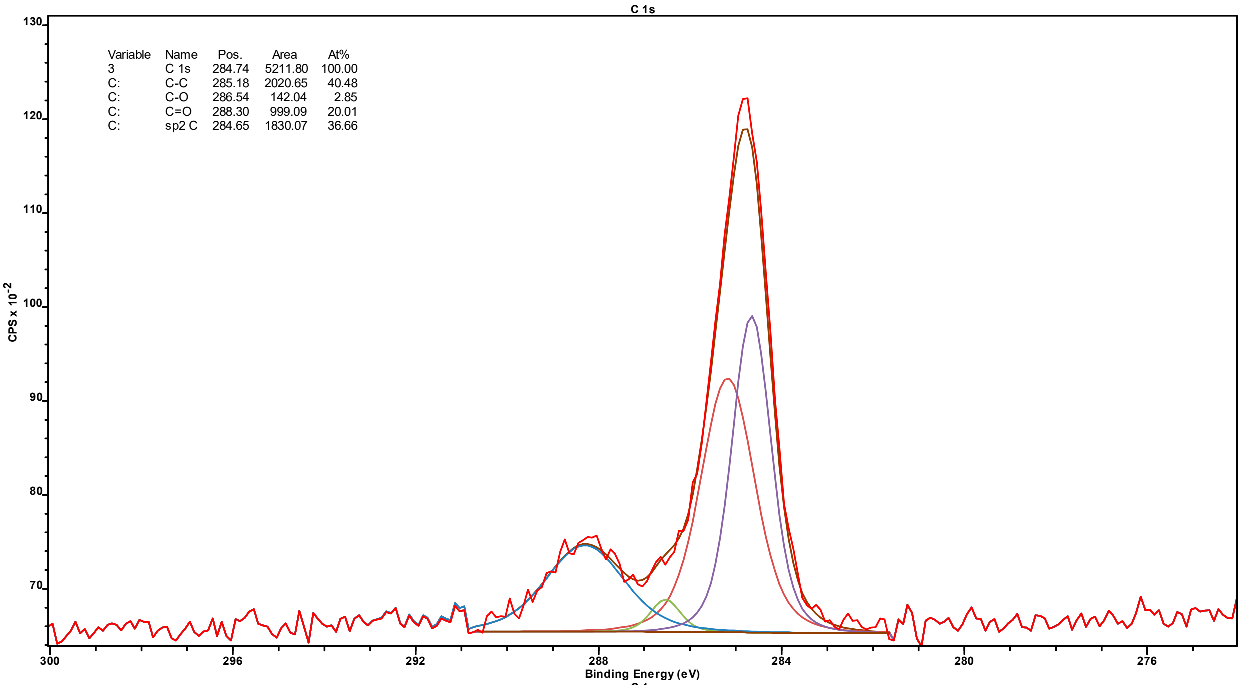
C 1s
Variable Name Pos. Area At%
3 C 1s 284.74 5211.80 100.00
C: C-C 285.18 2020.65 40.48
C: C-O 286.54 142.04 2.85
C: C=O 288.30 999.09 20.01
C: sp2 C 284.65 1830.07 36.66
CPS x 10-2
Binding Energy (eV)

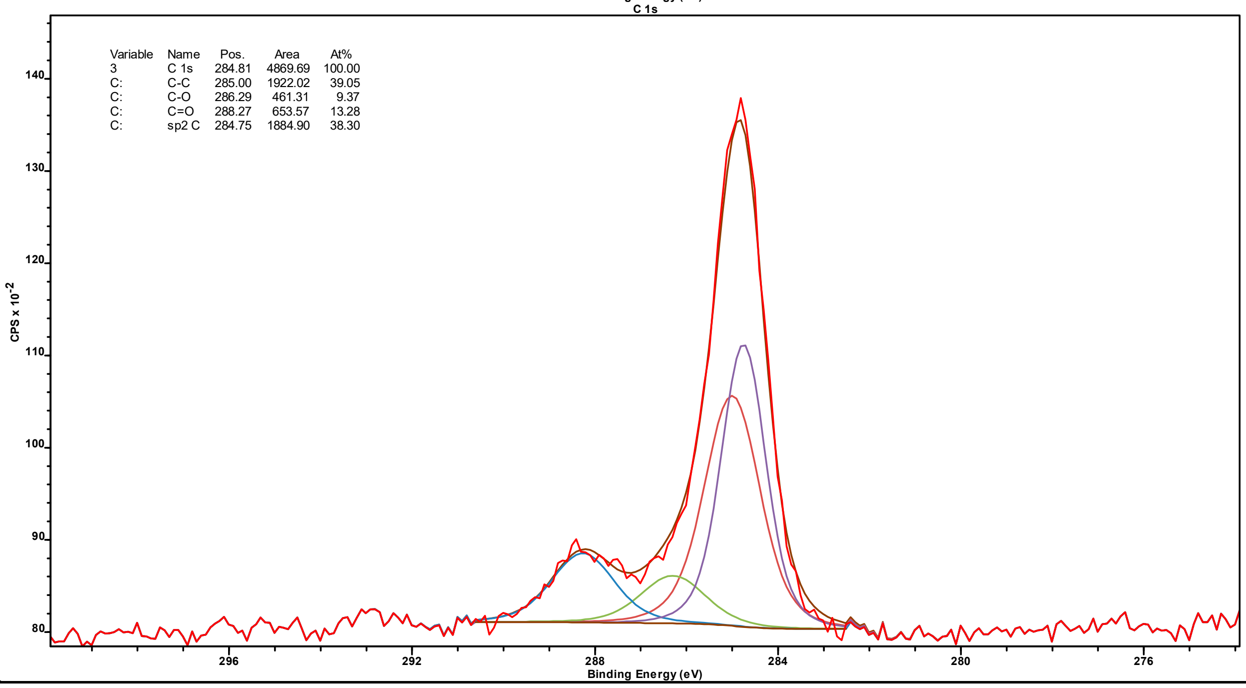
C 1s
Variable Name Pos. Area At%
3 C 1s 284.81 4869.69 100.00
C: C-C 285.00 1922.02 39.05
C: C-O 286.29 461.31 9.37
C: C=O 288.27 653.57 13.28
C: sp2 C 284.75 1884.90 38.30
CPS x 10-2
Binding Energy (eV)

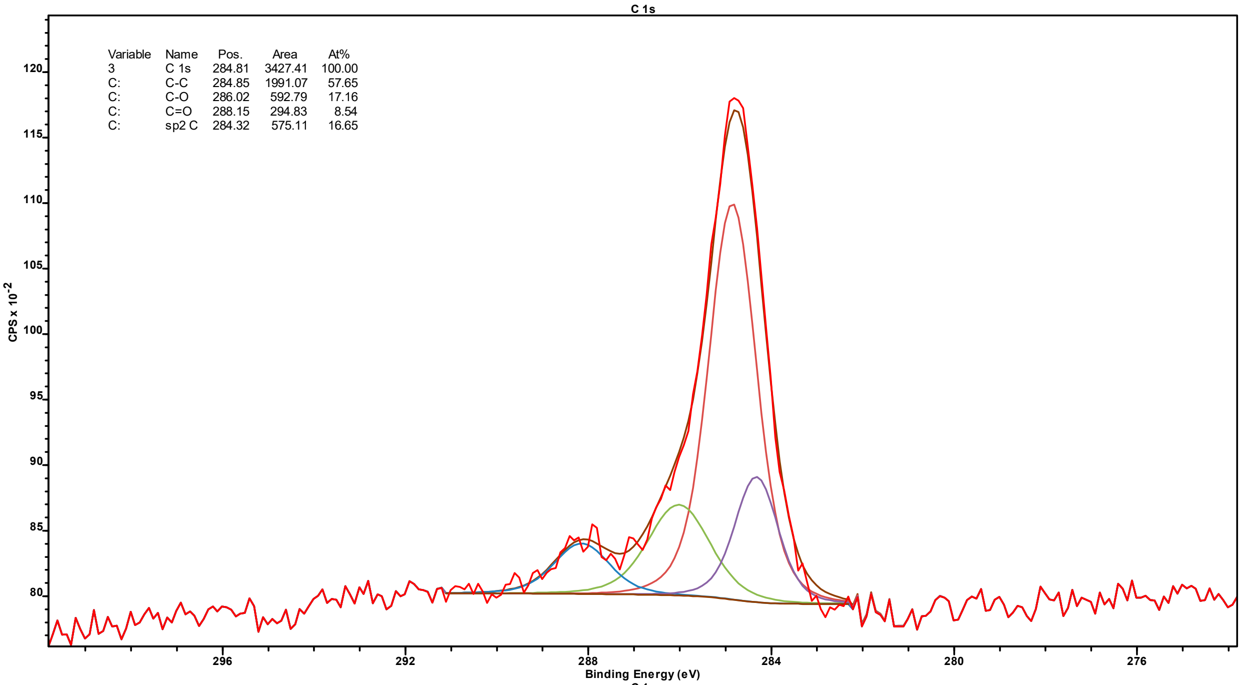
C 1s
Variable Name Pos. Area At%
3 C 1s 284.81 3427.41 100.00
C: C-C 284.85 1991.07 57.65
C: C-O 286.02 592.79 17.16
C: C=O 288.15 294.83 8.54
C: sp2 C 284.32 575.11 16.65
CPS x 10-2
Binding Energy (eV)

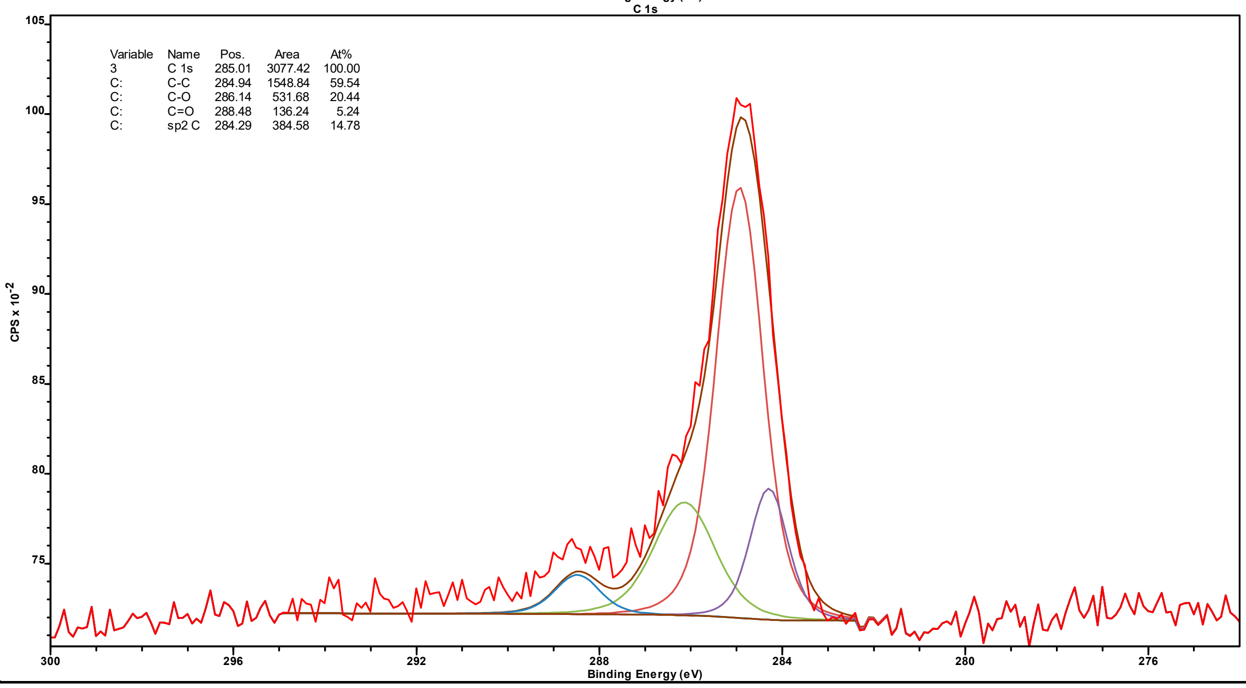
C 1s
Variable Name Pos. Area At%
3 C 1s 285.01 3077.42 100.00
C: C-C 284.94 1548.84 59.54
C: C-O 286.14 531.68 20.44
C: C=O 288.48 136.24 5.24
C: sp2 C 284.29 384.58 14.78
CPS x 10-2
Binding Energy (eV)

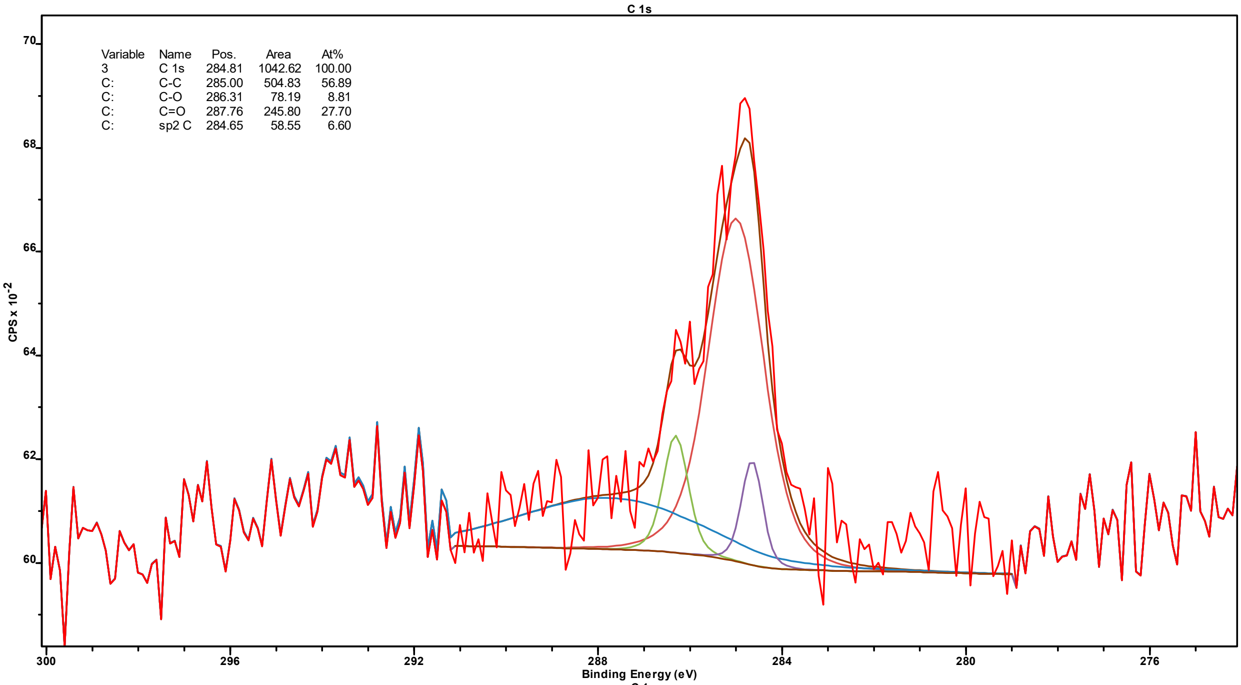
C 1s
Variable Name Pos. Area At%
3 C 1s 284.81 1042.62 100.00
C: C-C 285.00 504.83 56.89
C: C-O 286.31 78.19 8.81
C: C=O 287.76 245.80 27.70
C: sp2 C 284.65 58.55 6.60
CPS x 10-2
Binding Energy (eV)

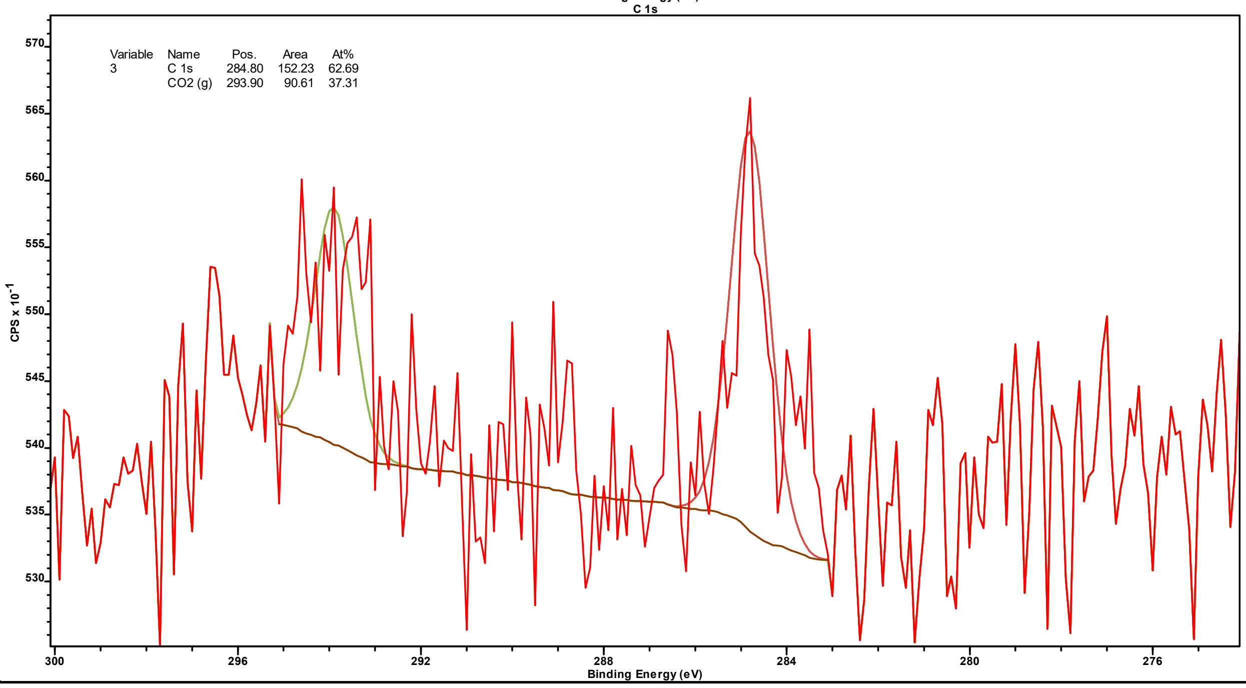
C 1s
Variable Name Pos. Area At%
3 C 1s 284.80 152.23 62.69
CO2 (g) 293.90 90.61 37.31
CPS x 10-1
Binding Energy (eV)

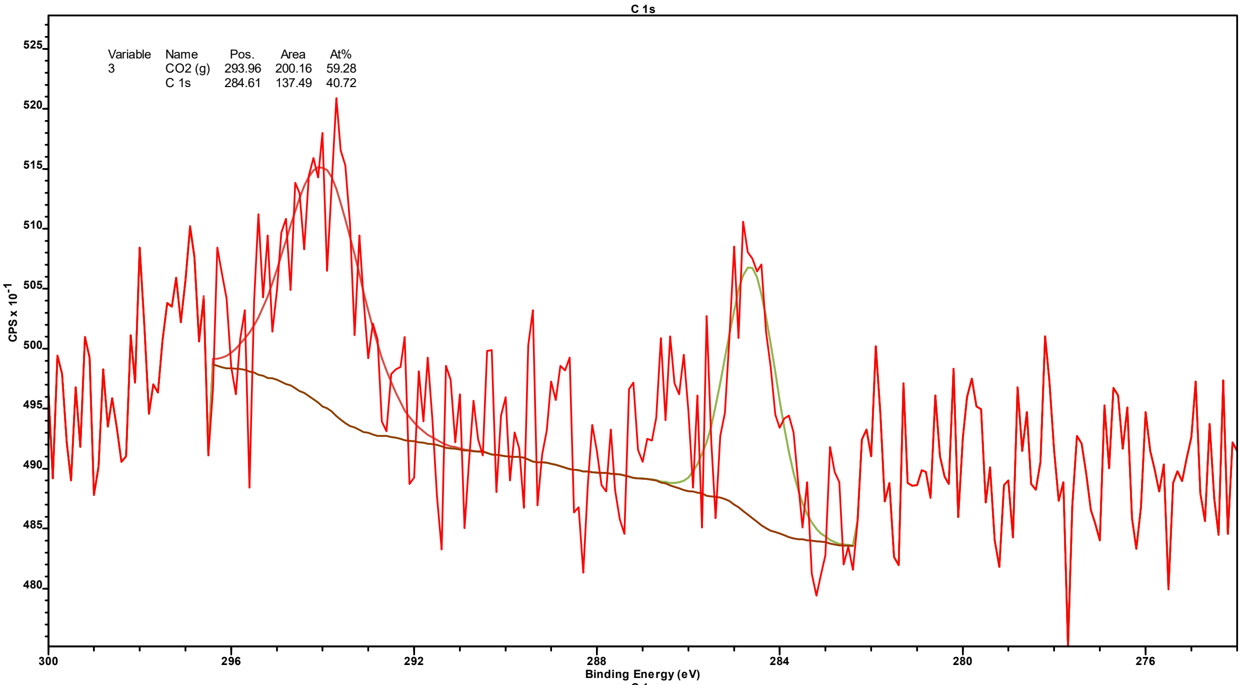
C 1s
Variable Name Pos. Area At%
3 CO2 (g) 293.96 200.16 59.28
C 1s 284.61 137.49 40.72
CPS x 10-1
Binding Energy (eV)

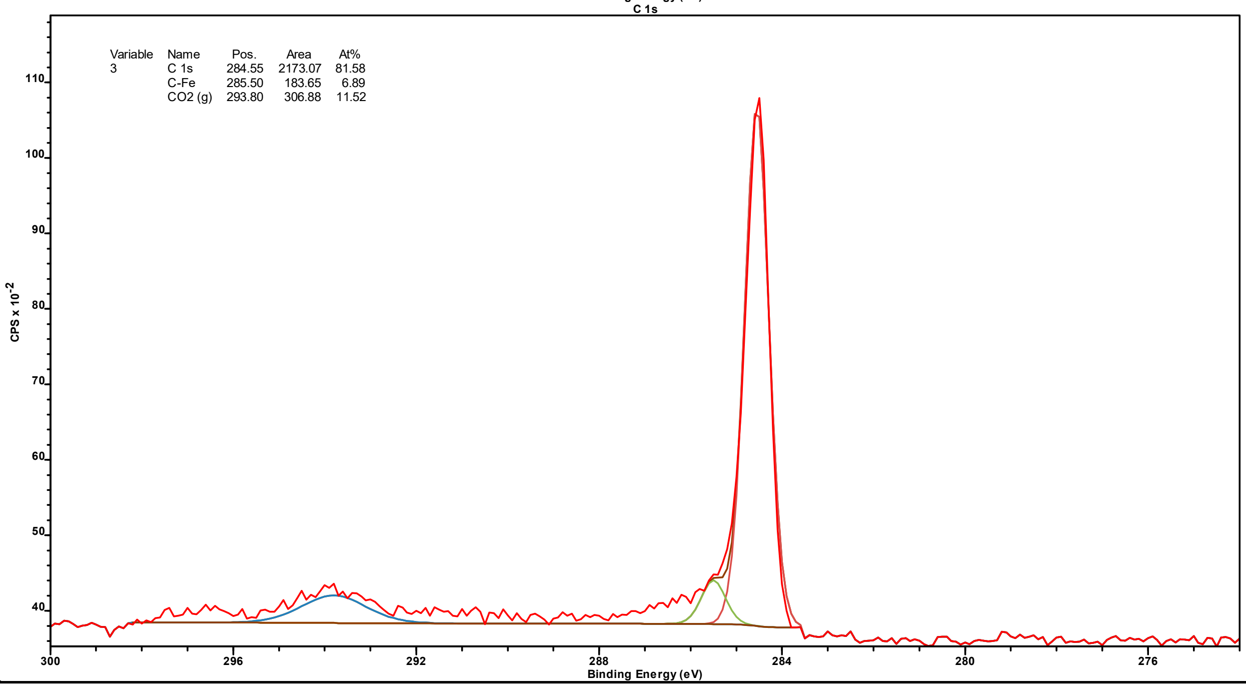
C 1s
Variable Name Pos. Area At%
3 C 1s 284.55 2173.07 81.58
C-Fe 285.50 183.65 6.89
CO2 (g) 293.80 306.88 11.52
CPS x 10-2
Binding Energy (eV)

873

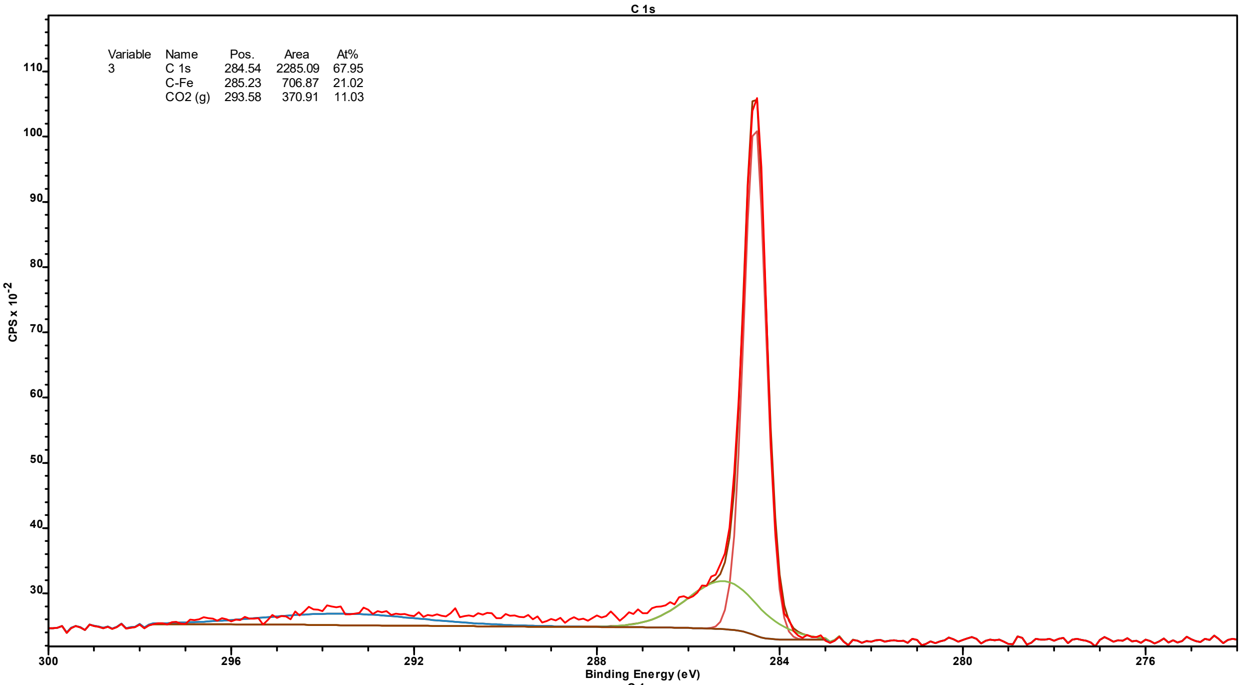
C 1s
Variable Name Pos. Area At%
3 C 1s 284.54 2285.09 67.95
C-Fe 285.23 706.87 21.02
CO2 (g) 293.58 370.91 11.03
CPS x 10^{-2}
110 100 90 80 70 60 50 40 30
300 296 292 288 284 280 276
Binding Energy (eV)

923

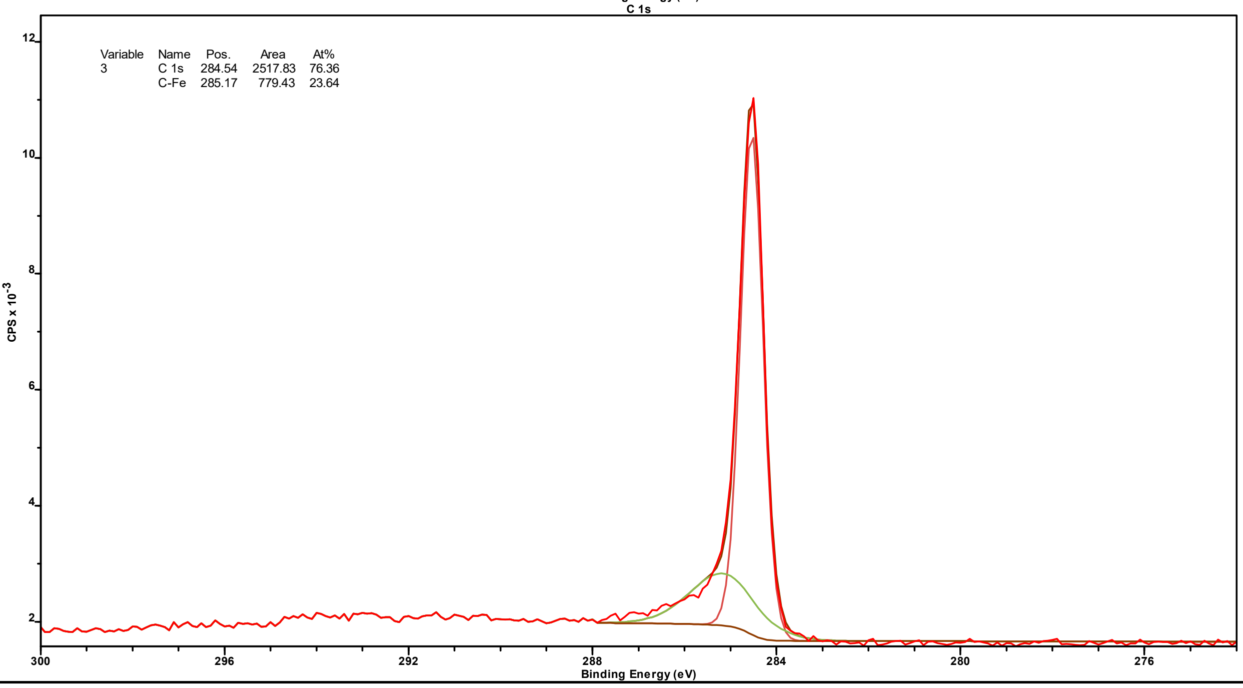
C 1s
Variable Name Pos. Area At%
3 C 1s 284.54 2517.83 76.36
C-Fe 285.17 779.43 23.64
CPS x 10^{-3}
12 10 8 6 4 2
300 296 292 288 284 280 276
Binding Energy (eV)
